# Acetyltransferase NAT10 regulates the Wnt/β-catenin signaling pathway to promote colorectal cancer progression via ac^4^C acetylation of KIF23 mRNA

**DOI:** 10.1186/s13046-022-02551-7

**Published:** 2022-12-15

**Authors:** Chi Jin, Tuo Wang, Dongsheng Zhang, Peng Yang, Chuan Zhang, Wen Peng, Kangpeng Jin, Lu Wang, Jiahui Zhou, Chaofan Peng, Yuqian Tan, Jiangzhou Ji, Zhihao Chen, Qingyang Sun, Sheng Yang, Junwei Tang, Yifei Feng, Yueming Sun

**Affiliations:** 1 Department of General Surgery, The First Affiliated Hospital of Nanjing Medical University, Nanjing, Jiangsu 210029 People’s Republic of China; 2 The First School of Clinical Medicine, Nanjing Medical University, Nanjing, China; 3 The Colorectal Institute of Nanjing Medical University, Nanjing, China

**Keywords:** Colorectal cancer, RNA modification, Ac^4^C, NAT10, KIF23, Wnt/β-catenin pathway, Remodelin

## Abstract

**Background:**

N^4^-acetylcytidine (ac^4^C) as a significant RNA modification has been reported to maintain the stability of mRNA and to regulate the translation process. However, the roles of both ac^4^C and its ‘writer’ protein N-acetyltransferase 10 (NAT10) played in the disease especially colorectal cancer (CRC) are unclear. At this point, we discover the underlying mechanism of NAT10 modulating the progression of CRC via mRNA ac^4^C modification.

**Methods:**

The clinical significance of NAT10 was explored based on the TCGA and GEO data sets and the 80 CRC patients cohort of our hospital. qRT-PCR, dot blot, WB, and IHC were performed to detect the level of NAT10 and ac^4^C modification in CRC tissues and matched adjacent tissues. CCK-8, colony formation, transwell assay, mouse xenograft, and other in vivo and in vitro experiments were conducted to probe the biological functions of NAT10. The potential mechanisms of NAT10 in CRC were clarified by RNA-seq, RIP-seq, acRIP-seq, luciferase reporter assays, etc.

**Results:**

The levels of NAT10 and ac^4^C modification were significantly upregulated. Also, the high expression of NAT10 had important clinical values like poor prognosis, lymph node metastasis, distant metastasis, etc. Furthermore, the in vitro experiments showed that NAT10 could inhibit apoptosis and enhance the proliferation, migration, and invasion of CRC cells and also arrest them in the G2/M phase. The in vivo experiments discovered that NAT10 could promote tumor growth and liver/lung metastasis. In terms of mechanism, NAT10 could mediate the stability of KIF23 mRNA by binding to its mRNA 3’UTR region and up-regulating its mRNA ac^4^c modification. And then the protein level of KIF23 was elevated to activate the Wnt/β-catenin pathway and more β-catenin was transported into the nucleus which led to the CRC progression. Besides, the inhibitor of NAT10, remodelin, was applied in vitro and vivo which showed an inhibitory effect on the CRC cells.

**Conclusions:**

NAT10 promotes the CRC progression through the NAT10/KIF23/GSK-3β/β-catenin axis and its expression is mediated by GSK-3β which forms a feedback loop. Our findings provide a potential prognosis or therapeutic target for CRC and remodelin deserves more attention.

**Graphical Abstract:**

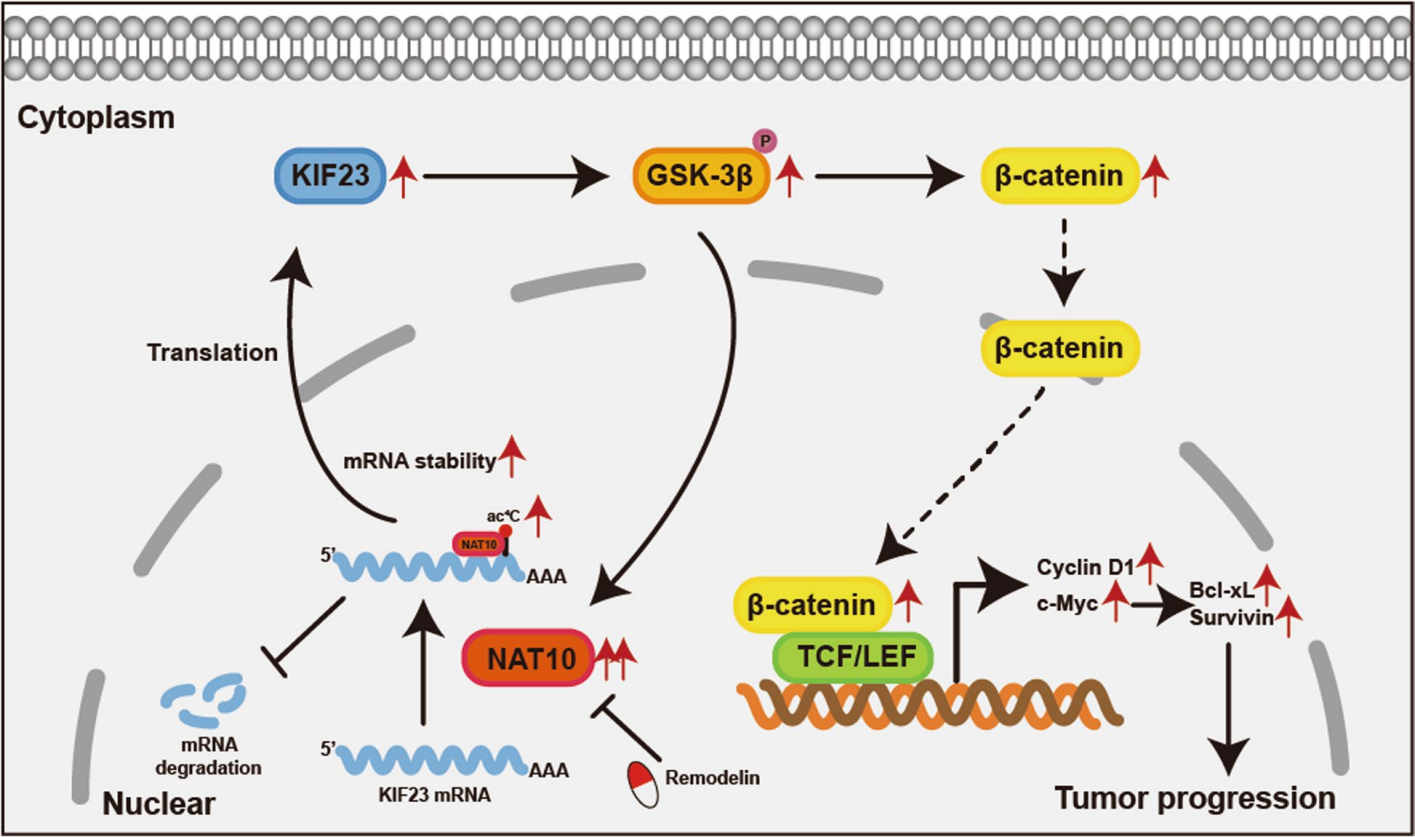

**Supplementary Information:**

The online version contains supplementary material available at 10.1186/s13046-022-02551-7.

## Introduction

Globally, colorectal cancer (CRC) has always been viewed as one of the most malignant tumors, with its incidence, particularly in developed countries, ranking third in both males and females, according to the most recent statistics on cancer. It is also worth noting that globally and relative to other cancer types, by 2022, its mortality rose to second place [1]. Specifically, in China, CRC ranks second in terms of incidence rate and fourth in terms of mortality rate [2], and despite the emergence of targeted therapy and immunotherapy in addition to traditional therapies, such as surgical excision, its prognosis remains poor given that it is characterized by rapid growth and distant invasion and metastasis. Therefore, to improve its treatment, there is an urgent need for the identification of potential molecular targets or genetic regulatory networks.

RNA modifications play a crucial role in molecular function regulation, and increasing evidence suggests that the pathways modulated by RNA modification might be potential targets for cancer therapy [3]. RNA modifications, including m^1^A, m^5^C, m^6^A, m^6^Am, m^7^G, and ac^4^C, mainly function to modulate RNA stability and translational efficiency [4–6], and reportedly, these regulations of RNA epigenetics are related to malignant biological properties in several cancer types [7–10]. However, studies on the role of ac^4^C, a highly conserved RNA modification that was first identified in 2018 on mRNA in individuals with some diseases, are limited [11]. It has been confirmed that ac^4^C modification is involved in several biological processes, including osteogenesis, AIDS, myocardial infarction, and cancers [8, 12–15]. However, previous studies on cancers in this regard have been limited to gastric and bladder cancers [8, 12]. Thus, ac^4^C is still unclear whether plays a vital role in CRC and whether it could be considered a promising target in the genetic therapy of CRC.

Mechanistically, previous studies have shown that RNA modifications depend on the binding to related proteins, such as METTL3, YTHDF1, and NSUN2 [16–18]. N-acetyltransferase 10 (NAT10), as the only protein that simultaneously contains an N-acetyltransferase domain and a nucleotide binding region, is recognized as the ac^4^C ‘writer’ protein that modulates the RNA modification process. As previously reported, NAT10 mainly acts as an acetyltransferase depending on the G641 in its N-acetyltransferase domain or as an E3 ligase which could regulate tRNA or rRNA acetylation [19–22]. As the only currently known ‘writer’ protein for ac^4^C modification, the importance of NAT10, about its involvement in cancers, especially those that exhibit its facilitatory effects, such as gastric cancer, bladder cancer, hepatocellular carcinoma, multiple myeloma, and non-small cell lung cancer, has been increasing highlighted [8, 12, 23–25]. However, the role of NAT10 in CRC seems to be controversial and needs further verification [19, 26, 27]. Besides, the Wnt/β-catenin pathway which is responsible for tumor progression might be correlated with NAT10, however, their relationship concerning CRC remains largely unknown [28].

Therefore, in this study, we elucidated the association between NAT10 expression and the clinicopathological characteristics of patients with CRC and for the first time, clarified the role of NAT10 in ac^4^C modification in CRC. Further, our study demonstrated that up-regulated NAT10 expression might lead to CRC progression via the acetylation of KIF23 mRNA and the further activation of the Wnt/β-catenin pathway. These findings, clarify the relationship between NAT10 and the Wnt/β-catenin pathway in CRC for the first time and the results of the successful treatment of CRC cells in vitro and in vivo with remodelin implied that NAT10 has potential value in targeted CRC therapy.

## Methods

### Clinical specimens and tissue microarray (TMA)

Colorectal tissue specimens, including tumor and adjacent tissues, were obtained from 80 patients who underwent resection surgery for CRC at the First Affiliated Hospital of Nanjing Medical University from 2017 to 2018. Written consents were approved by the 80 patients and the Human Ethics Committee of First Affiliated Hospital of Nanjing Medical University approved this study. All samples were collected during the surgery and were immediately frozen in liquid nitrogen or fixed in 4% formalin. A formalin-fixed paraffin-embedded (FFPE) tissue microarray was constructed by Servicebio (Wuhan, China) containing clinical specimens from 80 patients. The pathological information of the 80 patients was also obtained for further analysis and was provided in Table S1.

### Cell lines and culture

HEK293T, human colonic epithelial cell line NCM460 and human colorectal cancer cell lines SW480, DLD-1, HT-29, RKO, and HCT116 were purchased from Procell Life Science & Technology Co., Ltd (Wuhan, China) and the Cell Bank of Type Culture Collection of the Chinese Academy of Sciences (Shanghai, China) respectively. The cells were routinely cultured in the recommended medium with 10% fetal bovine serum and 1% penicillin/streptomycin at 37℃ in a 5% humidified and abacterial incubator.

### Western blot (WB) and antibodies

Proteins were extracted from cells according to the manufactory’s protocol (KeyGEN BioTech). The BCA Protein Assay Kit (Beyotime Biotechnology) was used to quantify the protein concentrations in the cell lysates. WB was performed as reported before [29]. The primary antibodies used are listed in Table S2.

### Plasmid construction and lentiviral infection

To be used in lentivirus-mediated interference or overexpression, the plasmids containing short hairpin RNAs (shRNAs) or full-length targeting sequence of NAT10 were synthesized by Tsingke (Beijing, China) and Obio (Shanghai, China) respectively. Besides, KIF23 shRNAs were synthesized by Tsingke (Beijing, China). The wild-type and mutant expression plasmids of NAT10 with a FLAG tag and KIF23 with a HA tag were constructed by Obio. Lipofectamine 3000 (Invitrogen, USA) was used to transfect cells with plasmids for transient transfection. For stable transfection, the infectious lentivirus particles were produced as described previously [30]. The sequences of shRNAs are listed in Table S3.

### RNA extraction and quantitative real-time PCR (qRT-PCR)

The specific procedures were performed according to a previous study [31]. The primers used in the study are listed in Table S3.

### Nuclear and cytoplasmic extraction

The nuclear and cytoplasmic fractions of CRC cells were isolated by using the PARIS™ kit (Thermo, USA). The procedure was performed according to the protocol of the manufacturer.

### Cell proliferation, transwell, and wound healing assays

The CCK-8 (Beyotime, China), colony formation, EdU (Beyotime, China), transwell, and wound healing assays were performed according to previous studies [32, 33].

### Flow cytometry assays of cell cycle and apoptosis

The flow cytometry assays were used to evaluate the distribution of the cell cycle and apoptotic rate of CRC cells. The specific procedures were performed as previously described [33].

### Dot blot

Procedures were similar to the previous study [7]. In brief, the diluted RNA was spotted to the Hybond-N + membrane (GE health, USA) after RNA extraction. After that, RNAs on the spotted membrane were UV cross-linked, blocked, and incubated with an ac^4^C antibody (Abcam). The membrane was subsequently incubated with horseradish peroxidase-conjugated anti-rabbit IgG secondary antibody and an ECL Western Blotting Detection Kit (Thermo Fisher Scientific) was used to visualize the signals from the dot blots.

### RNA-seq

shControl-SW480, shNAT10-SW480, shControl-DLD-1, and shNAT10-DLD-1 cells were used for the RNA-seq analysis. The procedure of RNA-seq was performed as previously described [33]. All data analysis and processing were performed by BGI (Shenzhen, China). The raw data is available in the GEO with the number GSE210384.

### RNA immunoprecipitation (RIP) and RIP-seq

The RNA immunoprecipitation (RIP) assay was performed according to the protocol of the Magna RIP Kit (Millipore, USA). Briefly, 5 μg NAT10 or FLAG antibodies and 50μL magnetic beads were well mixed and incubated with cell lysates. Then, RNAs were extracted after the removal of proteins. Followed by qPCR, the expression of genes was normalized to input. The process and analysis of RIP-seq in SW480 and DLD-1 cells were performed by Guangzhou Epibiotek Co., Ltd. (Guangzhou, China). The raw data could be found in GEO with the number GSE210385.

### Acetylated RNA immunoprecipitation (acRIP) and acRIP-seq

To quantify the level of ac^4^C modification of the specific gene, acetylated RNA immunoprecipitation (acRIP) was performed and the procedure was similar to the m^6^A RNA immunoprecipitation (MeRIP) assay by replacing the anti-m^6^A antibody with an anti-ac^4^C antibody according to the manufacturer’s instructions (Millipore, USA). In a nutshell, the anti-ac4C antibody (Abcam) was mixed with the beads overnight. Then, the complex was incubated with the RNA samples. After eluting the RNA from the beads, qPCR was performed. Besides, the service of acRIP-seq in SW480 and DLD-1 cells was provided by Guangzhou Epibiotek Co., Ltd. (Guangzhou, China). The raw data could also be found in GEO with the number GSE210385.

### RNA electrophoretic mobility shift assay (REMSA)

We amplified the NAT10-binding region on KIF23 mRNA by PCR with the T7 promoter sequence to generate the REMSA probes. Then, the biotin-labeled RNA probes were produced from the in vitro transcription according to the protocol of the MEGA Shortscript Kit (Ambion, USA). The REMSA was subsequently performed by using the LightShift Chemiluminescent RNA EMSA Kit (Thermo, USA). In brief, nuclear proteins in SW480 cells were extracted using a PARIS™ kit (Thermo, USA), and then the nuclear proteins were mixed with bio-labeled, unlabeled, or mutant RNA probes to form the RNA–protein complexes. The complexes were separated by 4% native polyacrylamide gel and transferred to a nylon membrane. After cross-linking the RNA to the membrane, it was then blocked and incubated with the chemiluminescent substrate buffer to expose. For the supershift assay, the NAT10 and IgG antibodies were added to the nuclear extracts respectively and the subsequent protocol used was the same as that above. The T7 promoter sequence and the sequences of probes for REMSA are listed in Table S3.

### Luciferase reporter assay

The promoter of KIF23 or the wide and mutant KIF23 3’UTR were inserted into the reporter plasmid. The luciferase reporter assay was performed following the protocol of the Dual-Luciferase reporter kit (Promega, USA). With the NAT10 knockdown or overexpression, the activities of firefly and Renilla luciferase were measured and the luciferase activities were normalized to Renilla fluorescence.

### RNA decay assay

NAT10-knockdown or NAT10-overexpression CRC cells were treated with actinomycin D (5 μg/mL) for 0, 4, 8, and 12 h(s). Then, total RNA was isolated to detect the relative levels of KIF23 by qPCR.

### Immunofluorescence (IF)

For the immunofluorescence (IF) assay, the procedure was described previously [26]. The antibodies used in the study are listed in Table S2.

### Immunohistochemistry (IHC)

The formalin-fixed paraffin-embedded (FFPE) tissue microarray was used for IHC analysis. The antibodies used in the study are listed in Table S2. The process of IHC staining was performed by Servicebio (Wuhan, China). The staining intensity (i) was estimated as weak (0), medium (1), strong (2), and strongest (3). The percentage of positively stained cells (pi) was divided into 0 ~ 5%, 6% ~ 25%, 26% ~ 50%, 51% ~ 75%, and 76% ~ 100%. Then, the histochemistry score (H-score) was used to evaluate the relative expression of genes. H-score = ∑ (pi × i).

### Animal models

Six-week-old male nude mice (BALB/c) mice divided into five per group were used for the xenograft tumor model and metastasis models. For the xenograft model, 100μL cell suspensions containing 1 × 10^6^ CRC cells stably transfected with shNAT10, shControl, oeNAT10, or oeVector were subcutaneously injected into the armpits of the mouse limbs. The tumor volumes were measured every 5 days. Twenty-five days after the injection, the mice were sacrificed to dissect the xenograft tumors and the weights of the tumors were measured. For metastasis models, 100μL cell suspensions containing 1 × 10^6^ CRC cells mentioned above were injected into the distal tip of the spleen or the tail veins of mice. Five weeks later, D-luciferin (150 mg/kg) (Goldbio, USA) was intra-peritoneal injected into the mice and the metastases were visualized using an IVIS 100 Imaging System (Xenogen, USA). After the imaging, the mice were excised under anesthesia to count the metastatic nodules in the lung or liver. To detect the inhibiting effect of remodelin on NAT10 in vivo, remodelin was used in xenograft tumor models of BALB/c nude mice by oral gavage at 100 mg/kg per day for 15 days from the 10^th^ day after the cell injection and in metastasis models by intraperitoneal injection at 5 mg/kg every other day for 4 weeks from the 1^st^ week after the cell injection. All animal experiments were performed under the experimental animal use guidelines of the National Institutes of Health.

### Statistical analysis

All data are presented as the mean ± standard error of the mean of at least three biological replicates. To perform the statistical analyses, GraphPad Prism 9.0 (La Jolla, USA) and SPSS 13.0 software (Chicago, USA) were used in this study. The student’s t-test analyzed the difference between the two samples while ANOVA was applied for more than two groups. Besides, the correlation among NAT10, KIF23, and β-catenin was analyzed by Pearson’s correlation analysis and Kaplan–Meier analysis was used to estimate the overall survival rates. The chi-square test was also performed to make clear the association between the levels of NAT10, KIF23, or β-catenin and clinicopathological characteristics of CRC patients. Among all analyses, *p* < 0.05 was considered statistically significant.

## Results

### NAT10 expression and the level of ac^4^C modification are significantly up-regulated in CRC

**Fig. 1:**
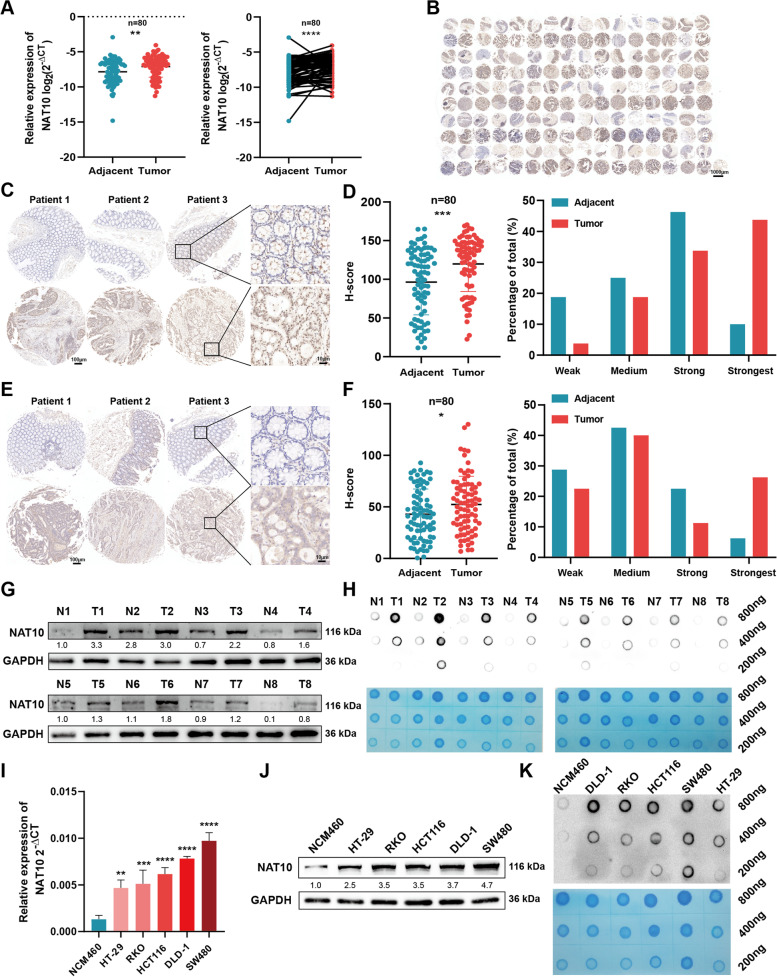
NAT10 and the level of ac^4^C modification are frequently upregulated in CRC. **A** The mRNA level of NAT10 detected by qRT-PCR in 80 CRC tissues and matched adjacent tissues. **B**-**D** The protein level of NAT10 detected by IHC in the TMA of 80 CRC patients’ samples. **E** and **F** The level of ac^4^C modification detected by IHC in the TMA. **G** The detection of the protein level of NAT10 by WB in 8 matched CRC tumor and adjacent tissues. **H** Level of ac.^4^C modification in 8 paired CRC tumor and adjacent tissues, as determined by dot blot. **I**-**K** The mRNA and protein levels of NAT10 and the level of ac4C in CRC cell lines and NCM460 cells, as determined by qRT-PCR, WB and dot blot respectively. Data are shown as mean ± SD of three independent experiments, *P < 0.05, **P < 0.01, ***P < 0.001, ****P < 0.0001

TO reflect the importance of NAT10 in CRC, we first measured NAT10 mRNA levels in 80 CRC tissue samples and their paired adjacent normal tissues via qRT-PCR (Fig. 1A, Table S5). Thus, we observed that the tumor tissues showed higher NAT10 expression levels, consistent with TCGA and GEO datasets (GSE41258) (Fig. S1A). Next, the tissue microarray (TMA) was prepared from the 80 patient samples to further explore the NAT10 protein level in CRC (Fig. S1C, D). Immunohistochemistry (IHC) in this regard showed up-regulated NAT10 expression in CRC tissues (Fig. 1B), as reflected by the higher degrees of staining and the H-scores obtained (Fig. 1C), consistent with Clinical Proteomic Tumor Analysis Consortium (CPTAC) data (Fig. S1B). Further, an exploration of the relationship between NAT10 protein level and the clinicopathological characteristics of patients with CRC showed that tumor stage, lymph node metastasis, vascular invasion, and distant metastasis were statistically significantly associated with NAT10 expression (Table 1). Specifically, clinical subgroup analysis demonstrated that the N1 + N2 group with lymph node metastasis, M1 group with distant metastasis, and III + IV tumor stage group showed higher NAT10 expression levels, implying that NAT10 can regulate CRC proliferation and metastasis (Fig. S1E). Furthermore, we detected ac^4^C levels by performing IHC staining on our TMA (Fig. S1F). Notably, consistent with NAT10 expression, the ac^4^C level was also dramatically upregulated in CRC tissues (Fig. 1E, F). To better reflect NAT10 protein level in this regard, we chose eight samples from the patients’ cohort to perform western blotting (WB), which showed that NAT10 was highly-expressed in CRC samples (Fig. 1G). The eight samples were also used for dot blotting, which also showed high ac^4^C levels in the tumor tissues (Fig. 1H). In addition to the detection of NAT10 and ac^4^C levels in tissues, our results also indicated that the levels of NAT10 and ac^4^C were likewise overexpressed in CRC cell lines (Fig. 1I-K). The analysis of all the CRC tissues and cell lines via immunofluorescence (IF) staining indicated that NAT10 was localized in the nucleus and cytoplasm of CRC cells, but predominantly in the nucleus (Fig. S1G, H). Taken together, these results revealed that the ac^4^C writer, NAT10 is up-regulated in CRC and is significantly associated with the clinicopathological characteristics of patients with CRC.

**Table 1:** Relevance analysis of NAT10 expression in CRC patients

**Varible**	**All patients**	**NAT10**		***P*** ** value**
		**High**	**Low**	
All Cases	80	40	40	
Age (years)				
60	23	15	8	0.084
≥ 60	57	25	32	
Gender				
Male	50	22	28	0.166
Female	30	18	12	
Tumor site				
Colon	35	21	14	0.115
Rectum	45	19	26	
Tumor size (cm)				
5	49	26	23	0.491
≥ 5	31	14	17	
TNM staging system				
T1 + T2	33	13	20	0.112
T3 + T4	47	27	20	
Tumor stage				
Stage I + II	43	9	34	** < 0.001** ^*^
Stage III + IV	37	31	6	
Lymph node metastasis				
No	46	11	35	**< 0.001** ^*^
Yes	34	29	5	
Vascular invasion				
No	61	25	36	**0.004** ^*^
Yes	19	15	4	
Nerve invasion				
No	66	30	36	0.078
Yes	14	10	4	
Distant metastasis				
No	63	27	36	**0.014** ^*^
Yes	17	13	4	
CEA (ng/ml)				
5	49	21	28	0.108
≥ 5	31	19	12	

The bold type represents *P* values smaller than 0.05

*TNM* Tumour node metastasis, *CEA* Carcinoembryonic antige

*P* < 0.05 was considered significant

### NAT10 enhances the proliferation, migration, and invasion of CRC cells in vitro

**Fig. 2:**
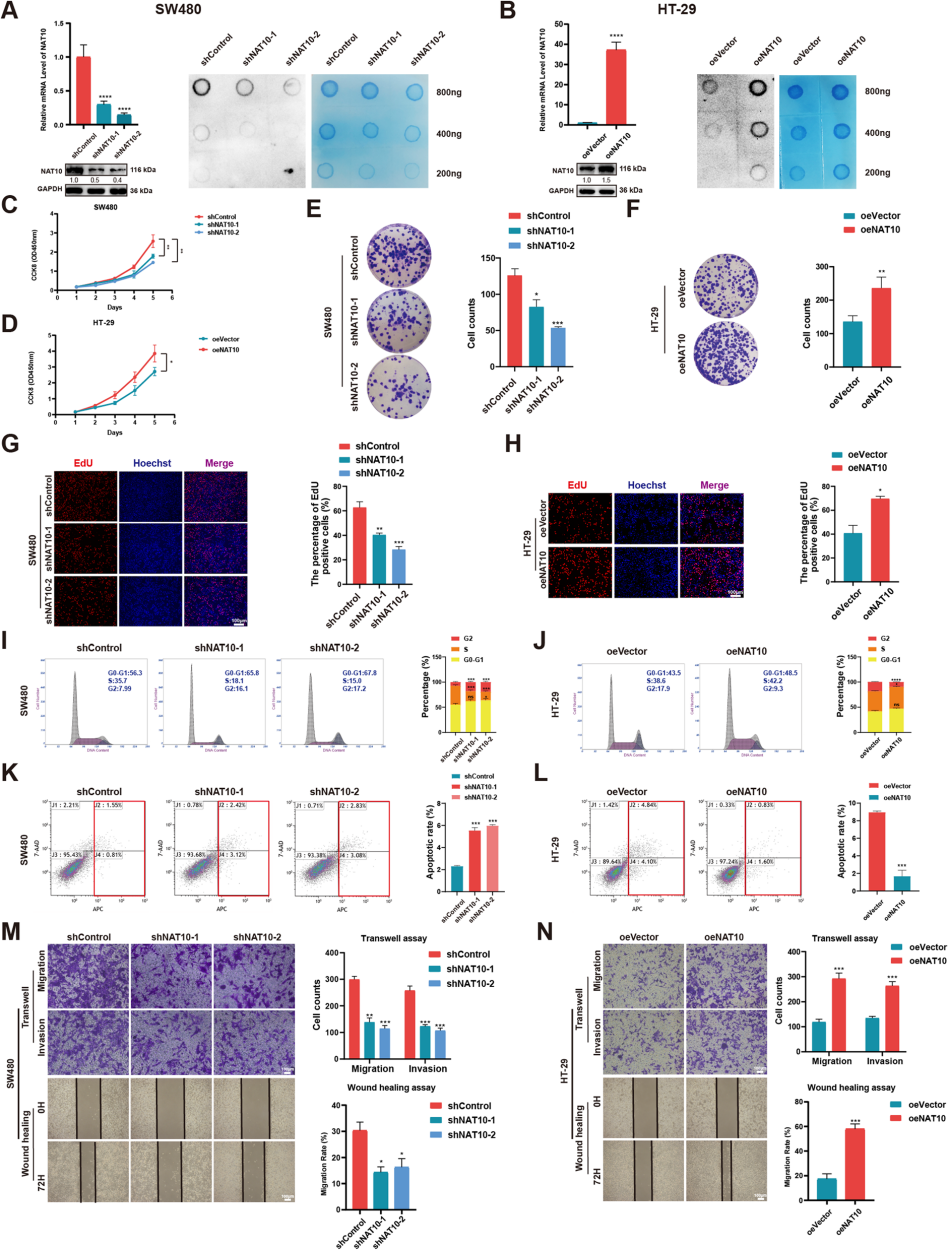
NAT10 promotes CRC cell proliferation, migration and invasion in vitro. **A** and **B** Transfection efficiency of NAT10 in SW480 and HT-29 cells, detected by qRT-PCR, WB, and dot blot. **C** and **D** CCK-8 assays were applied to determine the growth curves of NAT10 knockdown or overexpression cells. **E** and **F** Colony formation assays were carried out to detect the proliferation of CRC cells. **G** and **H** EdU assays were performed to evaluate the cell proliferation ability. **I** and **J** The distribution of the cell cycle was detected by flow cytometry in NAT10 knockdown or overexpression cells. **K** and **L** Cells were treated with the serum-free medium for 36 h. Flow cytometry was used to detect the apoptotic rates (LR + UR) of cells. **M** and **N** Transwell and wound healing assays were used to detect the migration and invasion of CRC cells. LR, early apoptotic cells; UR, terminal apoptotic cells. Data are shown as mean ± SD of three independent experiments, *P < 0.05, **P < 0.01, ***P < 0.001, ****P < 0.0001, ns. not significant

For verification using CRC cell lines, we specifically chose SW480 and DLD-1 cells, with high NAT10 expression, and HT-29 cells, with relatively low NAT10 expression. After the shNC, shNAT10-1, shNAT10-2, oeVector, and oeNAT10 plasmids were established, they were cloned into the lentivirus infection system to further construct stable cell lines. Transfection efficiency in the three cell lines mentioned above was then verified via qRT-PCR, WB, and dot blot (Fig. 2A, B, and Fig. S2A). Then, to evaluate the ability of NAT10 to adjust CRC cell proliferation, CCK-8, colony formation, and EdU assays were performed. Consistent with the assay results, NAT10 knockdown dramatically inhibited the proliferation of SW480 and DLD-1 cells, while its overexpression in HT-29 cells showed opposite effects (Fig. 2C-H and Fig. S2B-D). Additionally, it was evident that NAT10 knockdown arrested the growth of SW480 and DLD-1 cells in the G2/M phase and increased the ratio of apoptotic cells based on flow cytometric assays of cell cycle and apoptosis. Conversely, the number of CRC cells in the G2/M phase and the apoptotic rates were significantly decreased when NAT10 was overexpressed in HT-29 cells (Fig. 2I-L and Fig. S2E, F). Further, transwell and wound healing assays, performed to detect changes in cell migration and invasion abilities, showed that NAT10 depletion significantly impaired the migration and invasion abilities of SW480 and DLD-1 cells, while its overexpression led to contrary phenomena in HT-29 cells (Fig. 2M, N and Fig. S2G). Taken together, NAT10 could promote the proliferation of CRC cells and also regulate their migration and invasion.

### NAT10 facilitates the tumorigenesis and metastasis of CRC cells in vivo

**Fig. 3:**
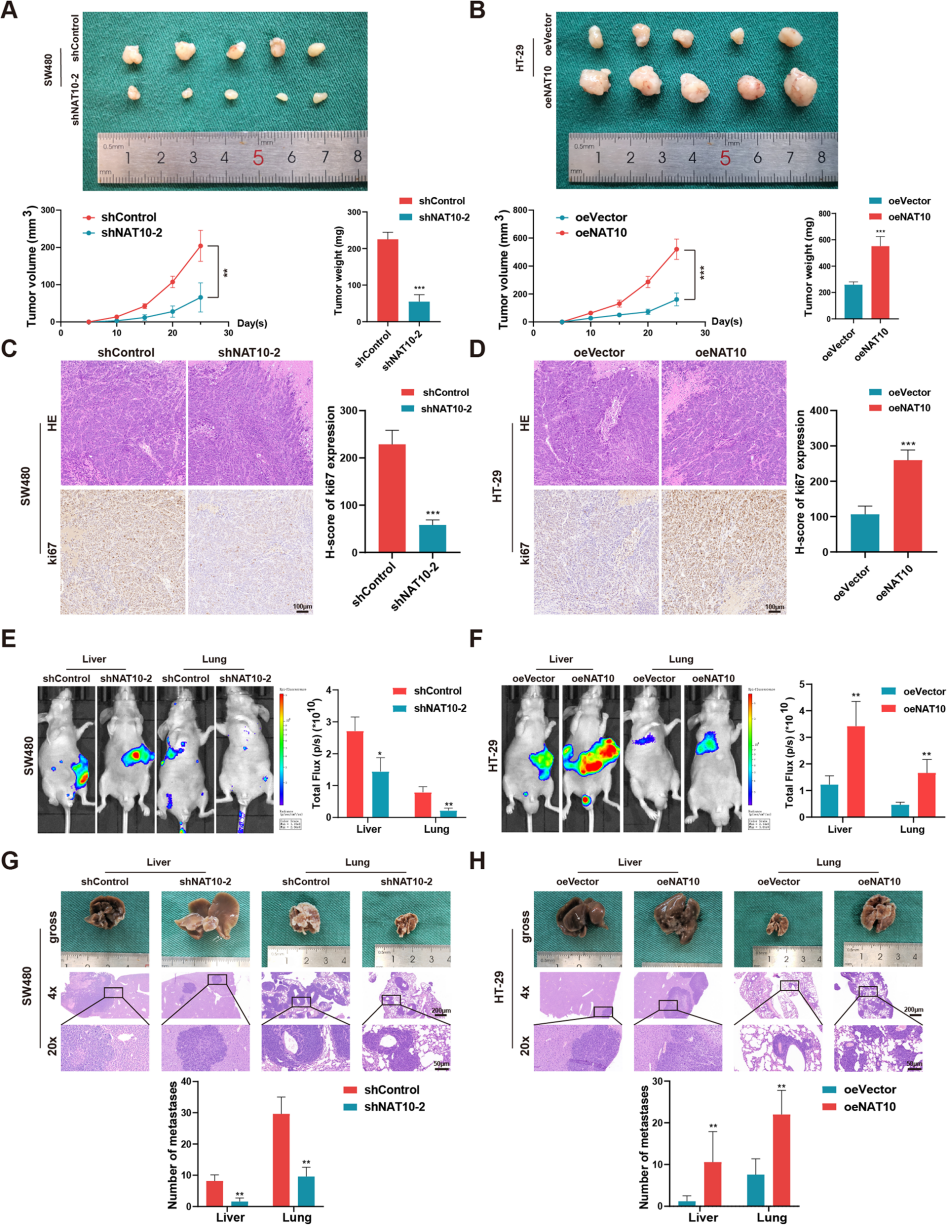
NAT10 facilitates tumor growth and metastasis in vivo. **A** and **B** Representative images of subcutaneous xenograft tumors (*n* = 5 for each group). The tumor volumes were measured every 5 days and the tumor weights were analyzed. **C** and **D** HE and IHC staining of xenograft tumors. The expression of Ki67 was detected by IHC. **E** and **F** Representative images and analysis of luminescence intensity in metastasis models (*n* = 5 for each group). **G** and **H** Representative image and HE staining of metastatic tumors in the livers and lungs of mice. The number of metastases in livers or lungs was analyzed. All data are presented as mean ± SD. *P < 0.05, **P < 0.01, ***P < 0.001

To explore the effect of NAT10 in vivo, xenograft tumor models and metastasis models were established. In the xenograft tumor model, SW480 and DLD-1 cells, stably transfected with shNC and shNAT10-2 and HT-29 cells stably transfected with oeVector and oeNAT10 were subcutaneously injected into nude mice. Thereafter, the analysis of changes in tumor weight and the trends of tumor volume revealed that NAT10 knockdown inhibited tumor growth in vivo, while its overexpression had an opposite effect (Fig. 3A, B and Fig. S3A). IHC showed that, compared with the control group, the shNAT10-2 group showed significantly downregulated Ki67 expression, while the oeNAT10 group showed significantly up-regulated Ki67 expression (Fig. 3C, D and Fig. S3B). Further, the liver and lungs of metastasis models were injected with stably transfected CRC cells via the distal tip of the spleen and tail veins of the nude mice, respectively. Thereafter, higher fluorescence intensity and larger numbers of metastasis nodules in the liver and lungs indicated that NAT10 facilitated metastasis, which could be restrained by its knockdown (Fig. 3E-H and Fig. S3C, D). Thus, the animal models revealed that CRC cell tumorigenesis and metastasis in vivo could be promoted by upregulated NAT10 expression, consistent with the findings obtained in vitro.

### Identification of the profile of ac^4^C-modified genes regulated by NAT10 in CRC cells

**Fig. 4:**
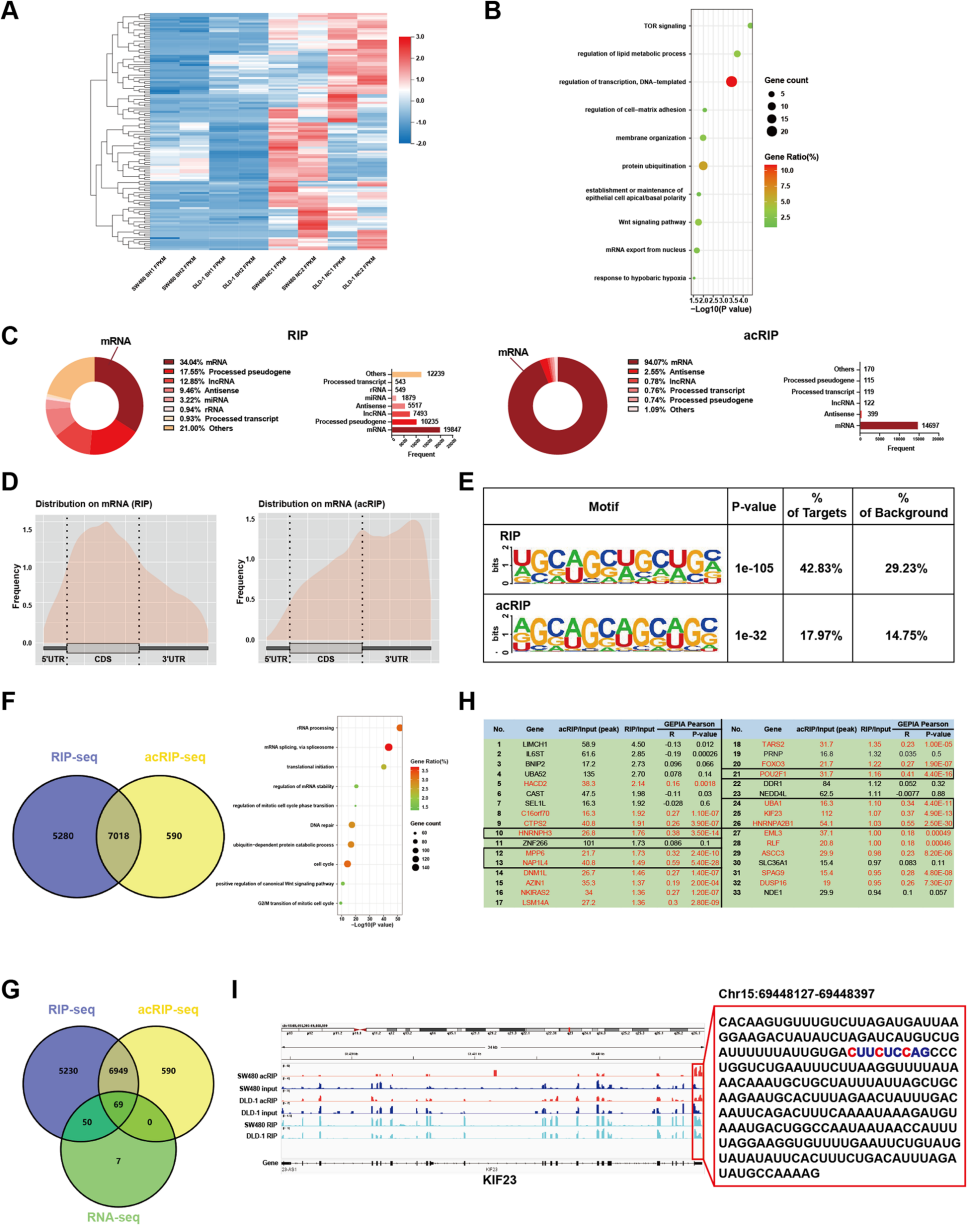
Identification of the NAT10 targets in CRC cells. **A** Heatmap of significantly down-regulated genes in NAT10 knockdown cells identified by RNA-seq. **B** GO enrichment analysis of the significantly down-regulated genes. **C** Distribution of NAT10 targeted transcripts identified by RIP-seq and acRIP-seq. **D** The distribution of NAT10-binding regions and ac^4^C peaks across mRNA in CRC cells. **E** The consensus sequences of NAT10-binding sites and ac^4^C motif were detected by the motif analysis with RIP-seq and acRIP-seq data. **F** GO enrichment analysis of the genes overlapped from the genes identified by RIP-seq and acRIP-seq. **G** Overlapping analysis of genes identified by RNA-seq, RIP-seq, and acRIP-seq. H. The list of the potential direct targets of NAT10. **I** Distribution of NAT10-binding regions and ac4C peaks on KIF23 mRNA visualized by IGV

To determine how NAT10 regulated CRC progression at the transcriptional level, we first subjected NAT10-knockdown and control SW480 and DLD-1 cells to RNA-seq. Thereafter, differentially expressed genes (fold change > 1.20 or < 0.83, *p* < 0.05) between the two groups of SW480 and DLD-1 cells were shown using volcano plots (Fig. S4A), while the down-regulated genes in CRC cells upon the knockdown of NAT10 were shown using a heatmap (Fig. 4A). To explore the biological processes NAT10 might be involved in, 126 genes significantly downregulated and 58,232 genes altered by NAT10 in both SW480 and DLD-1 cells were respectively included for gene ontology (GO) analysis and gene set enrichment analysis (GSEA) (Fig. S4B, C). GO analysis showed that the enriched pathways included TOR signaling, protein ubiquitination, the Wnt signaling pathway, and mRNA export from the nucleus, while GSEA indicated that NAT10 might be associated with the CTNNB1 oncogenic signature as well as epithelial-mesenchymal transition (Fig. 4B and Fig. S4D).

Moreover, given that NAT10 mainly participates in epigenetic regulation by binding and affecting ac^4^C acetylated transcripts and to clarify its role as an ac^4^C ‘writer’ protein, we further performed RIP-seq and acRIP-seq using SW480 and DLD-1 cells (the acRIP-seq process is shown in Fig. S4E). Notably, RIP-seq revealed that NAT10 binds to 58,302 transcripts, among which 34.04% were mRNAs. Additionally, acRIP-seq showed 15,622 ac^4^C peaks corresponding to 7608 transcripts, 94.07% of which were observed in mRNA. This observation represented the main type of transcripts ac^4^C modification happened in and the potential combination of NAT10 with mRNA (Fig. 4C). After analyzing the distribution on mRNA, we noticed the appearance of NAT10-binding regions and ac^4^C peaks in coding sequences (CDS) and 3’-untranslated regions (3’-UTR), consistent with previously reported data [8, 12] (Fig. 4D). Motif analysis further indicated that the typical ac^4^C motif, ‘CXXCXXCXX’, was significantly enriched both in the NAT10-binding and ac^4^C-modified sequences, suggesting that NAT10 possibly modulated the ac^4^C modification of mRNA by binding to it (Fig. 4E). Based on GO analysis, we also observed that NAT10-binding (FPKM > 0 both in IP and IgG groups) and ac^4^C-modified genes were involved in rRNA processing, translational initiation, mRNA stability regulation, cell cycle, and the positive regulation of the canonical Wnt signaling pathway (Fig. 4F).

To further clarify the target genes and consider the crucial effects of NAT10 on mRNA stability, we overlapped the genes identified based on RNA-seq, RIP-seq, and acRIP-seq. Thus, we observed that 69 genes bound by NAT10 were tagged with ac^4^C and were down-regulated upon NAT10 knockdown (Fig. 4G). Thereafter, by re-ranking the abovementioned 69 genes via fold-enrichment in RIP-seq and acRIP-seq followed by overlapping the top two-thirds of them considering the two datasets, 33 genes were identified as potential NAT10 direct targets (Fig. S4F). Furthermore, the correlation of these 33 genes’ expression with NAT10 was observed using the public GEPIA dataset based on the TCGA (http://gepia.cancer-pku.cn). Based on the correlation analysis, the expression of 21 genes was notably positively correlated with NAT10, with only seven genes’ (*HNRNPH3, MPP6, NAP1L4, POU2F1, UBA1, KIF23, HNRNPA2B1*) showing Pearson correlation coefficients > 0.3 (Fig. 4H). Next, using IGV, we visualized the obvious ac^4^C peaks and NAT10-binding peaks corresponding to these seven genes and via qRT-PCR, observed the mRNA levels of these genes in 24 randomized patient samples. As shown (Fig. 4I and Fig. S4G, H), KIF23 changed markedly in tumor tissues corresponding to adjacent tissues among the 7 candidate genes and the potential ac^4^C motif in the modified region (chr15:69 448 127–69 448 397) on KIF23 mRNA 3’UTR region might be ‘CUUCUCCAG’. Therefore, we successfully identified the profile of ac^4^C-modified genes regulated by NAT10 in CRC, with KIF23 seeming to be a direct target of NAT10 in CRC cells.

### NAT10 stimulates KIF23 expression via ac^4^ C modification

**Fig. 5:**
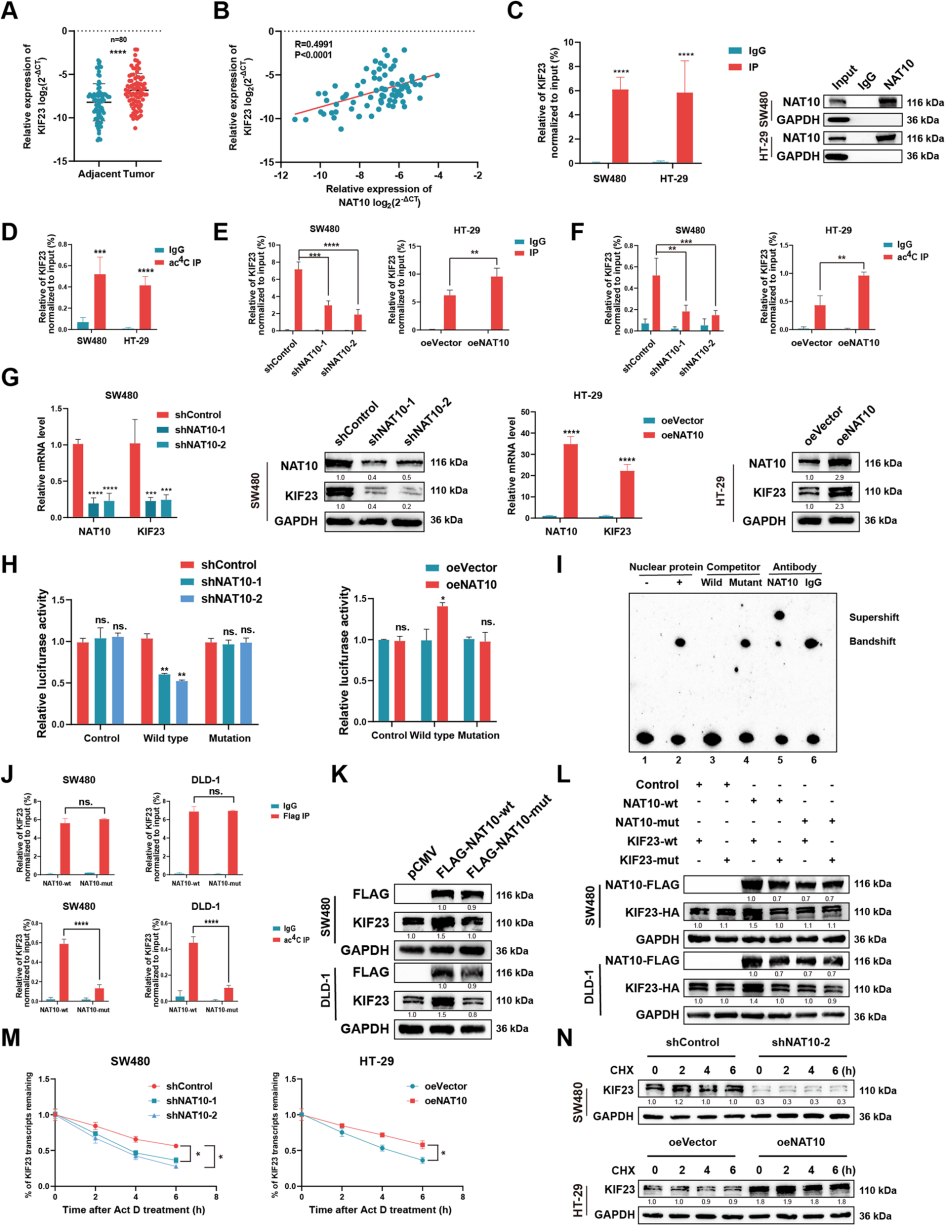
NAT10 stimulates the expression of KIF23 through ac^4^C modification. **A** The mRNA level of KIF23 detected by qRT-PCR in 80 CRC tissues and matched adjacent tissues. **B** Correlation analysis between the mRNA levels of NAT10 and KIF23. **C** NAT10 RIP followed by qPCR in SW480 and HT-29 cells. **D** acRIP followed by qPCR in SW480 and HT-29 cells. **E** The interaction between NAT10 and KIF23 mRNA was analyzed by RIP-qPCR assay in CRC cells with NAT10 knockdown or overexpression. **F** The relative levels of ac^4^C in KIF23 were tested by acRIP-qPCR in CRC cells with NAT10 knockdown or overexpression. **G** Relative RNA and protein level of KIF23 in CRC cells upon NAT10 knockdown or overexpression. **H** The luciferase activity for the reporter containing the NAT10-binding region or mutant upon NAT10 knockdown or overexpression. **I** REMSA assays to detect the combination of NAT10 and the ac^4^C motif on KIF23 mRNA. **J** RIP and acRIP followed by qPCR upon the transfection of NAT10-wt or NAT10-mut in CRC cells. **K** The expression of KIF23 upon overexpression of Flag-tagged NAT10 wide-type or its mutant, as determined by WB in SW480 and DLD-1 cells. **L** The expression of KIF23 in SW480 or DLD-1 cells co-transfected with empty vector, wild-type or mutant Flag-tagged NAT10, and wild-type or mutant HA-tagged KIF23. **M** The mRNA stability was detected by qRT-PCR in SW480 and HT-29 cells with the addition of actinomycin D (5 μg/mL). **N** The protein expression of KIF23 with the treatment of CHX (100 μg/mL) in CRC cells upon NAT10 knockdown or overexpression. Data are shown as mean ± SD of three independent experiments, *P < 0.05, **P < 0.01, ***P < 0.001, ****P < 0.0001, ns. not significant

To explore the correlation between the expression levels of NAT10 and KIF23, we first detected the KIF23 mRNA level of KIF23s in tissue samples from 80 patients with CRC via qRT-PCR (Table S5). Thus, we observed higher KIF23 mRNA levels in tumor tissues as well as a positive correlation between KIF23 mRNA expression and NAT10, consistent with TCGA or GEO datasets (GSE40967) (Fig. 5A, B and Fig. S5A). Next, the detection of KIF23 protein levels via IHC using the TMA showed similar results implying the existence of a potential regulatory association between NAT10 and KIF23 (Fig. S5B, C). We also noted that KIF23 protein expression was significantly associated with tumor site, tumor stage, lymph node metastasis, nerve invasion, and CEA among the 80 patient samples (Table 2). The verification of the interaction between NAT10 and KIF23 mRNA via RIP-qPCR assays using three CRC cell lines showed a notable enrichment of NAT10 in conjunction with KIF23 mRNA compared with the IgG groups (Fig. 5C and Fig. S5D). Consistent with the results of acRIP-seq, acRIP followed by qPCR confirmed the abundance of ac^4^C modifications on KIF23 mRNA (Fig. 5D and Fig. S5E). Further, we observed that the interaction between NAT10 and KIF23 mRNA and the abundance of ac^4^C modification sites on KIF23 mRNA could be changed following NAT10 knockdown and overexpression, which contributed to stronger binding between NAT10 and KIF23 mRNA and up-regulated ac^4^C modification in KIF23 mRNA. Furthermore, NAT10 disruption led to opposite results (Fig. 5E, F, Fig. S5F, G). Considering the correlation between NAT10 and KIF23, we further investigated KIF23 expression via qRT-PCR and WB upon NAT10 knockdown and overexpression. The same expression change tendencies were observed for all three CRC cell lines (Fig. 5G and Fig. S5H). Then, to demonstrate that the regulatory effect of NAT10 on KIF23 could be primarily attributed to its binding to the 3’-UTR region of KIF23 mRNA rather than direct or indirect regulation of the promoter activity, two experimental scenarios were established. In the first scenario, we noticed that the luciferase activity for a reporter containing the KIF23 promoter region remained unchanged upon NAT10 knockdown or overexpression in CRC cells, indicating that NAT10 could not regulate KIF23 expression by directly or indirectly modulating its promoter activity of KIF23 (Fig. S5I). In the second scenario, we investigated whether NAT10 could specifically bind to the ac^4^C-modified regions in the 3’-UTR region of KIF23 mRNA via luciferase reporter assay with the reporter containing the ac^4^C-modified regions of the 3’-UTR region. The wild type contained the potential ac^4^C-modified regions, while the mutation type did not. Thus, we observed that the luciferase activity of the reporter region containing the ac^4^C-modified regions could be repressed in SW480 and DLD-1 cells with NAT10 knockdown and enhanced in HT-29 cells with NAT10 overexpression. Conversely, the mutation group did not show any significant changes in this regard (Fig. 5H and Fig. S5J). To further confirm the direct binding between NAT10 and KIF23 mRNA, SW480 cells were subjected to RNA electrophoretic mobility shift assay (REMSA) which showed that the complex formed as a result of the reaction between the labeled probes and nuclear proteins could be inhibited by the unlabeled probes, and this inhibition could be attenuated by the mutant probes. The supershifted complex phenomenon was also observed after NAT10 antibody addition, indicating the direct binding between NAT10 with the ac^4^C motif in KIF23 mRNA (Fig. 5I and Fig. S5K).

**Table 2:** Relevance analysis of KIF23 expression in CRC patients

**Varible**	**All patients**	**KIF23**		***P*** ** value**
		**High**	**Low**	
All Cases	80	40	40	
Age (years)				
60	23	13	10	0.459
≥ 60	57	27	30	
Gender				
Male	50	26	24	0.644
Female	30	14	16	
Tumor site				
Colon	35	23	12	**0.013** ^*^
Rectum	45	17	28	
Tumor size (cm)				
5	49	25	24	0.819
≥ 5	31	15	16	
TNM staging system				
T1 + T2	33	13	20	0.112
T3 + T4	47	27	20	
Tumor stage				
Stage I + II	43	16	27	**0.014** ^*^
Stage III + IV	37	24	13	
Lymph node metastasis				
No	46	18	28	**0.024** ^*^
Yes	34	22	12	
Vascular invasion				
No	61	28	33	0.189
Yes	19	12	7	
Nerve invasion				
No	66	28	38	**0.003** ^*^
Yes	14	12	2	
Distant metastasis				
No	63	28	35	0.056
Yes	17	12	5	
CEA (ng/ml)				
5	49	18	31	**0.003** ^*^
≥ 5	31	22	9	

The bold type represents P values smaller than 0.05

*TNM* Tumour node metastasis, *CEA* Carcinoembryonic antige

*P* < 0.05 was considered significant

Given that KIF23 expression was altered in conjunction with changes in NAT10 expression, we confirmed the bind of NAT10 to KIF23 mRNA via its ac^4^C motif. Next, we wondered whether KIF23 regulation by NAT10 was ac^4^C dependent. Reportedly, NAT10 functions as an acetyltransferase, owing to its N-acetyltransferase domain (558–753) and the mutation in G641 can abrogate its acetyl-CoA binding structure [22]. Subsequently, we introduced a point G641E mutation in the N-acetyltransferase domain of NAT10 with FLAG tag (NAT10-mut) or NAT10 wide type (NAT10-wt) and transfected them into SW480 and DLD-1 cells (Fig. S5L). Interestingly, RIP and acRIP followed by qPCR revealed that NAT10-mut could impair ac^4^C modification on KIF23 mRNA, but not affect the bond between NAT10 and KIF23 mRNA (Fig. 5J). We also observed that NAT10-wt, but not NAT10-mut, could upregulate the expression of KIF23, suggesting that the N-acetyltransferase domain of NAT10 played an important role in mediating ac^4^C modification (Fig. 5K). In addition, the HA-tagged KIF23 expression vector (KIF23-wt) and its mutant, with mutations ac^4^C sites (KIF23-mut), were constructed (Fig. S5M), and as expected, we demonstrated that NAT10-wt, but not NAT10-mut, could upregulate KIF23-wt expression and that NAT10-wt could not influence KIF23-mut expression due to the absence of the ac^4^C motif (Fig. 5L).

Reportedly, NAT10 might regulate the stability of mRNA or its translation efficiency to modulate gene expression [11, 13]. Thus, we treated CRC cells with actinomycin D (5 μg/mL) to examine RNA decay following NAT10 knockdown or overexpression. The results obtained indicated that NAT10 could enhance KIF23 mRNA stability, and this effect could subsequently enhance protein translation (Fig. 5M and Fig. S5N). Previous studies have indicated that NAT10 can acetylate histones via its acetyltransferase activity or promote protein degradation via its E3 ligase activity [19]. To exclude the possibility that NAT10 might mediate the stability of KIF23 protein, we treated CRC cells with the protein translation inhibitor cycloheximide (CHX) (100 μg/mL) and observed a mild effect on KIF23 protein stability (Fig. 5N and Fig. S5O). Collectively, these results indicated that NAT10 regulated KIF23 mRNA via ac^4^C modification.

### NAT10 regulates the Wnt/β-catenin pathway via the NAT10/KIF23/GSK-3β axis

**Fig. 6:**
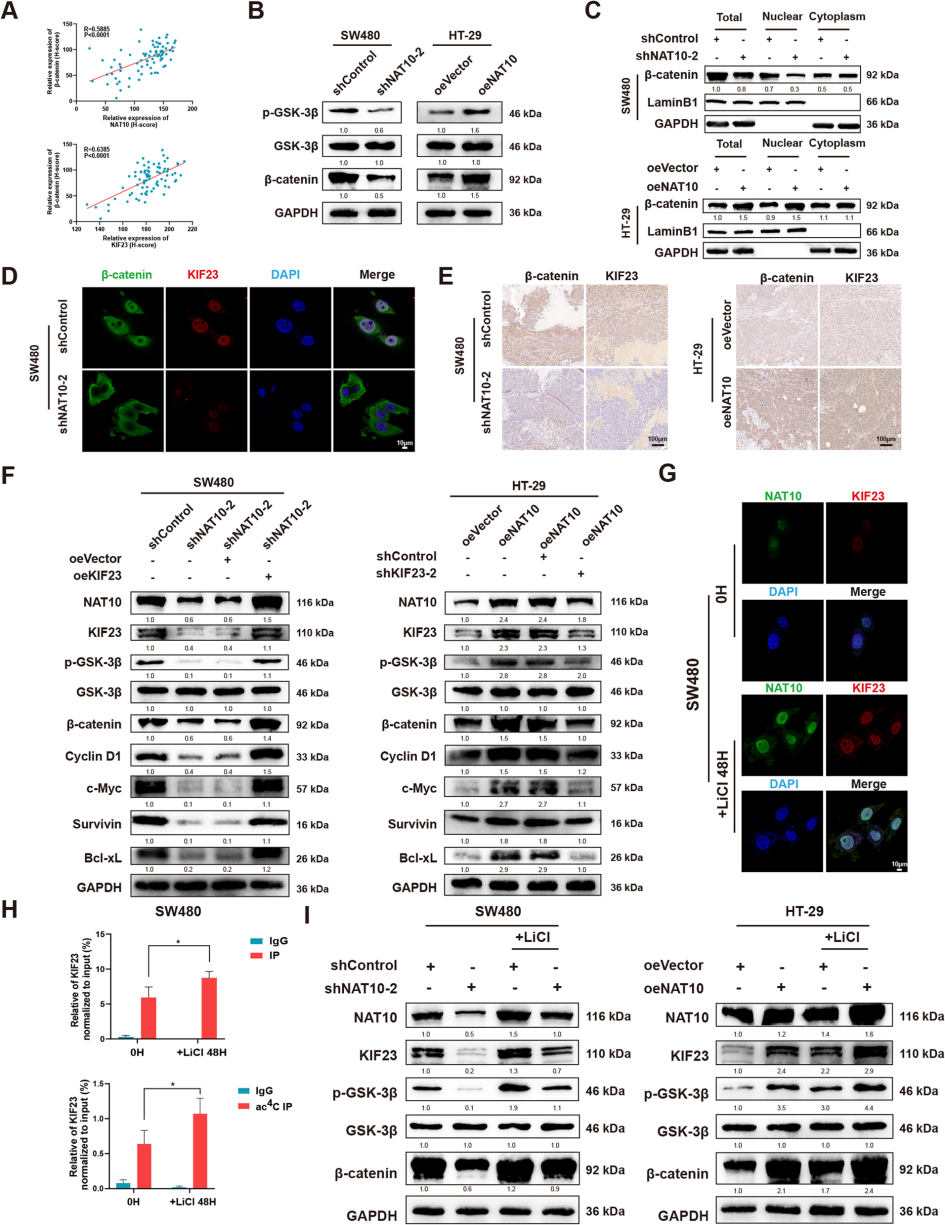
The NAT10/KIF23 axis regulates CRC cells and GSK-3β/NAT10/KIF23/GSK-3β loop participants in the progression. **A** Correlation analysis between the protein level of β-catenin and NAT10 or KIF23 detected by IHC. **B** The expression of GSK-3β, phosphorylated GSK-3β and β-catenin were determined by WB upon NAT10 knockdown or overexpression in CRC cells. **C** The level of β-catenin in the cell nucleus and cytoplasm was determined by WB in SW480 and HT-29 cells. **D** β-catenin nuclear translocation and KIF23 were detected by IF staining upon NAT10 knockdown in SW480 cells. **E** IHC staining of xenograft tumors. The expression of β-catenin and KIF23 were detected by IHC. **F** The expression of GSK-3β, phosphorylated GSK-3β, and β-catenin along with NAT10, KIF23, and the downstream genes of β-catenin (Cyclin D1, c-Myc, Survivin, and Bcl-xL) were detected by WB in relatively treated cells. **G** NAT10 and KIF23 were detected by IF staining with or without the treatment of LiCl (20 mmol/L) in SW480 cells for 48 h. **H** RIP and acRIP followed by qPCR with or without the treatment of LiCl (20 mmol/L) in SW480 cells for 48 h. **I** The expression of NAT10, KIF23, GSK-3β, phosphorylated GSK-3β, and β-catenin was detected by WB with or without the treatment of LiCl (20 mmol/L) in SW480 and HT-29 cells for 48 h. Data are shown as mean ± SD of three independent experiments, *P < 0.05

TO clarify the downstream of the KIF23 pathway, we performed GSEA analysis using TCGA and GEO datasets (GSE4097), which demonstrated the importance of NAT10 to cell cycle, G2/M checkpoint, MYC targets, and Wnt/β-catenin signaling (Fig. S6A). Considering previous studies, which showed that KIF23 exerts a regulatory effect on the Wnt/β-catenin pathway and the significant correlation between NAT10 and β-catenin [34–36], we hypothesized that NAT10 possibly regulates the Wnt/β-catenin pathway by mediating KIF23. Next, the detection of β-catenin expression in the CRC TMA showed high β-catenin expression, which was positively correlated with the expression levels of NAT10 and KIF23 according to the Pearson correlation analysis (Fig. 6A and Fig. S6B). β-catenin protein expression level also showed a strong correlation with tumor site, TNM staging system, tumor stage, lymph node metastasis, and distant metastasis (Table S4). Subsequently, to investigate whether NAT10 modulates the Wnt/β-catenin pathway in CRC, we measured phosphorylated GSK-3β, GSK-3β, and β-catenin levels as a function of changes in NAT10 levels, and observed that the Wnt/β-catenin pathway could be notably activated by NAT10 (Fig. 6B and Fig. S6C). Moreover, the subcellular protein fraction assay confirmed that NAT10 knockdown impaired β-catenin expression in the nucleus, while its overexpression significantly enhanced β-catenin expression in the nucleus (Fig. 6C and Fig. S6D). IF assay using SW480 and DLD-1 cells and IHC using xenograft tumor tissues as mentioned above showed similar results (Fig. 6D, E and Fig. S6E, F). Additionally, after the transfection efficiency in CRC cells following KIF23 knockdown or overexpression was verified, a series of rescue experiments were performed in vitro (Fig. S6G). Thus, we observed that CRC cell proliferation abrogated by shNAT10-2 could be rescued by oeKIF23, while that enhanced by oeNAT10 could be decreased by shKIF23-2 (Figs. S6H, I and S7A). Furthermore, oeKIF23 could reverse G2/M arrest and the apoptotic rates caused by NAT10 knockdown, while shKIF23-2 could lead to G2/M arrest and increased apoptotic rates following NAT10 overexpression (Fig. S7B, C). Transwell and wound healing assays yielded similar results (Fig. S7D). Next, considering cell cycle regulation by NAT10, the transcriptional targets that were activated the most by β-catenin, including cyclin D1 and c-Myc, were detected via WB. Survivin and bcl-xl, downstream of c-Myc, acting as typical anti-apoptotic proteins, were found to induce G2/M transition. Our results also indicated that NAT10 knockdown significantly downregulated KIF23 protein levels of KIF23, phosphorylated GSK-3β, β-catenin, cyclin D1, c-Myc, surviving, and bcl-xl, while NAT10 upregulation yielded opposite results. In the co-transfected groups, sh-KIF23-2 could reverse the regulatory effects mediated by oeNAT10, and the effects caused by sh-NAT10-2 were rescued by oeKIF23 (Fig. 6F and Fig. S8A).

Interestedly, we noticed that NAT10 could be modulated by GSK-3β [26]. This observation raised our curiosity regarding the existence of a regulation loop. Following treatment with LiCl (20 mmol/L), a GSK-3β inhibitor, for 48 h, IF assays showed increasing NAT10 and KIF23 staining (Fig. 6G and Fig. S8B). RIP or acRIP after the LiCl treatment followed by qPCR also showed an obvious increase in KIF23 mRNA (Fig. 6H and Fig. S8C). Similarly, WB confirmed that GSK-3β inhibition could rescue the effects of shNAT10-2 on the NAT10/KIF23/GSK-3β/β-catenin axis and also enhance the up-regulatory effects of oeNAT10 (Fig. 6I and Fig. S8D). These observations indicated that in CRC cells, NAT10 regulates the Wnt/β-catenin pathway via the NAT10/KIF23/GSK-3β loop.

### Targeting NAT10 using remodelin exhibits potential therapeutic effects

**Fig. 7:**
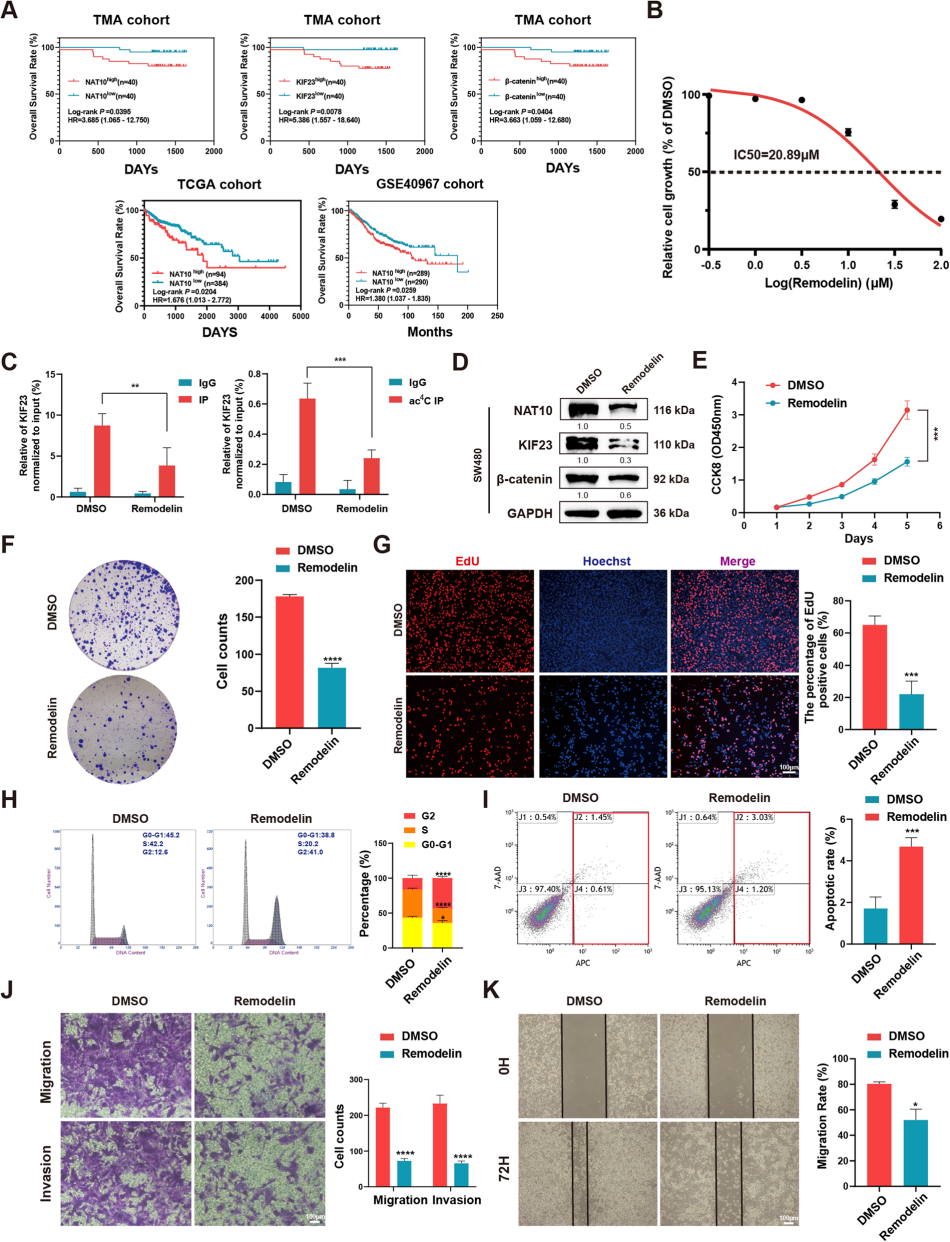
The NAT10 suppressor role of remodelin in CRC cells in vitro. **A** Effects of NAT10, KIF23, and β-catenin expression levels on CRC patient overall survival. **B** The IC50 value of remodelin was calculated in SW480 cells by CCK-8 assay. **C** RIP and acRIP followed by qPCR with the treatment of LiCl (20 mmol/L) or DMSO in SW480 cells for 48 h. **D** The expression of NAT10, KIF23, and β-catenin were detected by WB in SW480 cells with the treatment of LiCl (20 mmol/L) or DMSO for 48 h. **E**–**G** CCK-8, colony formation, and EdU assays were performed in SW480 cells with the treatment of LiCl (20 mmol/L) or DMSO for 48 h. **H** and **I** The flow cytometry of cell cycle and apoptosis were performed in SW480 cells with the treatment of LiCl (20 mmol/L) or DMSO for 48 h. **J** and **K** Transwell and wound healing assays were performed in SW480 cells with the treatment of LiCl (20 mmol/L) or DMSO for 48 h. Data are shown as mean ± SD of three independent experiments, *P < 0.05, **P < 0.01, ***P < 0.001, ****P < 0.0001

To investigate the clinical significance of NAT10, first, Kaplan–Meier analysis using data corresponding to 80 patients with CRC revealed that higher NAT10, KIF23, and β-catenin expression levels were associated with poorer overall survival (OS), and the analysis based on NAT10 was consistent with TCGA and GEO datasets (GSE40967) (Fig. 7A).

**Fig. 8:**
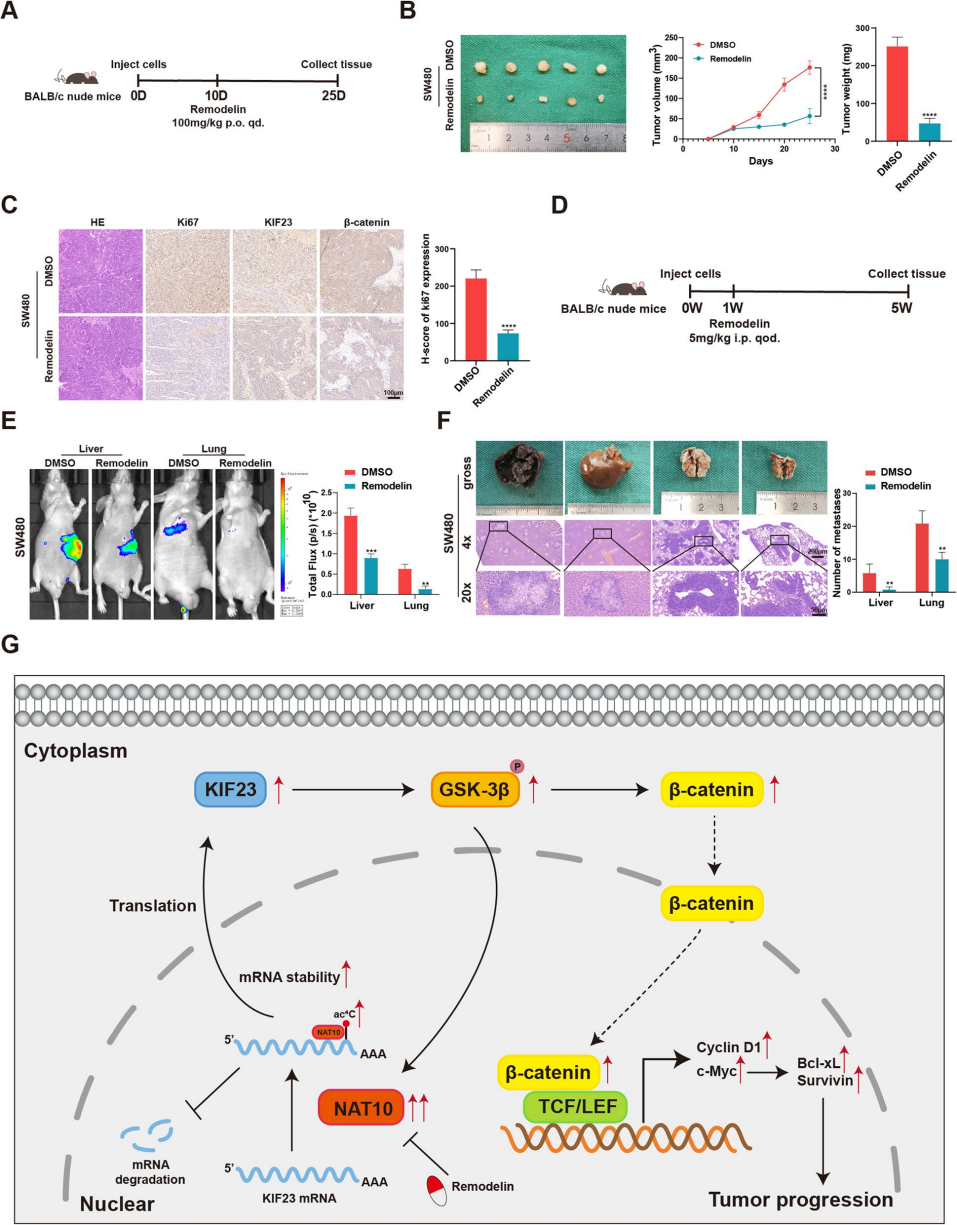
The role of remodelin in vivo and a schematic model for the mechanisms of NAT10. **A** The schematic diagram of the application of remodelin in the xenograft models of mice. **B** Representative images of subcutaneous xenograft tumors (*n* = 5 for each group). The tumor volumes were measured every 5 days and the tumor weights were analyzed. **C** HE and IHC staining of xenograft tumors. The expression of Ki67, KIF23, and β-catenin were detected by IHC. **D** The schematic diagram of the application of remodelin in the metastasis models of mice. **E** Representative images and analysis of luminescence intensity in metastasis models (*n* = 5 for each group). **F** Representative image and HE staining of metastatic tumors in the livers and lungs of mice. The number of metastases in livers or lungs was analyzed. **G** The schematic model for the mechanisms of NAT10 in CRC. All data are presented as mean ± SD. **P < 0.01, ***P < 0.001, ****P < 0.0001

Subsequently, remodelin, which was reported to be a chemical inhibitor of NAT10 [22, 37, 38], was applied to detect its inhibitory effect on NAT10 in vitro and vivo. By treating SW480 cells with remodelin at different concentrations, the half-maximal inhibitory concentration (IC50) of this NAT10 inhibitor in SW480 cells was 20.89 μM (Fig. 7B). Further, after remodelin (20 μM) treatment for 48 h, the ac^4^C modification on KIF23 mRNA and the bond between NAT10 and KIF23 mRNA were notably weakened (Fig. 7C). Furthermore, in SW480 cells the downstream of NAT10, including KIF23 and β-catenin, were found to be significantly downregulated by remodelin (Fig. 7D). In vitro, the proliferation, migration, and invasion abilities of SW480 cells were suppressed by remodelin, and SW480 cells also showed G2/M arrest and an increased rate of apoptosis following remodelin treatment (Fig. 7E-K). In vivo, xenograft tumor models of BALB/c nude mice were administered remodelin via oral gavage (100 mg/kg per day) for 15 days, while the metastasis models were administered remodelin via intraperitoneal injection at 5 mg/kg every other day for 4 weeks (Fig. 8A, D). The results thus obtained indicated that remodelin could notably inhibit the growth of xenograft tumors and the IHC analysis of the expression of Ki67, KIF23 and β-catenin presented similar results (Fig. 8B, C). Live imaging and the numbers of metastasis nodules in the liver and lungs of the model mice showed that remodelin could also significantly inhibit SW480 cells metastasis to the lungs or liver (Fig. 8E, F). All these findings demonstrated that NAT10 is a great prognostic indicator and targeting it using remodelin could inhibit CRC cell progression in vitro and in vivo, providing a potential prognosis or therapeutic target for CRC.

## Discussion

Given that several research methodology-related limitations have been overcome in the past few years, there has been a dramatic increase in the number of studies on RNA modifications. Benefiting from advanced high-throughput sequencing technologies, the importance of RNA modifications in diseases has been uncovered [39–41]. A growing body of research suggests that the maladjustment of RNA modification has considerable significance in cancer development [3, 42–44]. Among these RNA modifications, m^6^A has received the most attention [45, 46], while others, including m^5^C, m^1^A, m^7^G, and ac^4^C only came to public attention in recent years owing to recent research advances.

First reported in the 1980s, studies showed that ac^4^C modification alters tRNA to maintain its stability [47, 48]. Along with the identification of the ac^4^C position in RNA [49], Daniel et al. [11] strikingly established the role of ac^4^C in mRNA regulation. Since then, several researchers have shown increasing interest on ac^4^C modification [50]. Further, NAT10, which simultaneously contains an N-acetyltransferase domain and a nucleotide binding region [22], has been recognized as the only ‘writer’ protein of ac^4^C to mediate the ac^4^C modification process. Even though it has been suggested that NAT10 is involved in ribosome biogenesis, aging syndrome, DNA damage, and cancers [20, 21, 51, 52], studies on the mechanism by which it modulates ac^4^C modification in cancer development, especially CRC, are limited. In relation to cancers, NAT10 reportedly promotes tumor progression in hepatocellular carcinoma, breast cancer, gastric cancer, head, and neck squamous cell carcinoma, and bladder cancer [8, 12, 24, 53, 54]. In CRC, Liu et al. reported that NAT10 could suppress CRC by acetylating p53 into executing its function [19]. Conversely, Zhang et al. identified NAT10 as an oncogene in CRC mediated by GSK-3β [26]. In our study, we observed that NAT10 regulation in CRC was associated with aberrant ac^4^C modification on KIF23 mRNA. The difference between our results of those of Lui et al. might be attributed to the p53 mutation, which has been observed in ~ 60% of CRC cases [55]. Coincidentally, the CRC cell lines used in this study, SW480, DLD-1, and HT-29, were all p53 mutant types, while the HCT116 cell line used by Liu et al. was the p53 wild type. This phenomenon has also been observed in hepatocellular carcinoma, indicating that NAT10 could enhance mutant p53 activity to promote hepatocellular carcinoma proliferation [56]. Thus, to be precise, the relationship between NAT10 and p53 in CRC needs to be further investigated.

Our study revealed that NAT10 expression was associated with tumor stage, lymph node metastasis, vascular invasion and distant metastasis in patients with CRC and a higher NAT10 expression level implied a poorer prognosis. We also observed that NAT10 could facilitate CRC cell proliferation, migration, and invasion in vitro and in vivo, but these effects could be inhibited by remodelin. To elaborate on the specific underlying mechanism, RNA-seq, RIP-seq, and acRIP-seq showed that KIF23 mRNA was a direct ac^4^C target of NAT10. Reportedly, KIF23 is the regulator of the central spindle assembly and is involved in the malignant behavior of several cancers via the Wnt/β-catenin signaling pathway [34–36, 57]. Further investigation also revealed that NAT10 could enhance KIF23 expression by binding to the ac^4^C motif on KIF23 mRNA and activating the downstream of the Wnt/β-catenin pathway, clarifying the relationship between NAT10 and Wnt/β-catenin signaling in CRC for the first time. Further, according to Zhang [26], NAT10 was not a characteristic target gene of β-catenin but it could truly be mediated by GSK-3β after our confirmation which forms the NAT10/KIF23/GSK-3β feedback loop. Interestingly, we identified c-Myc as the downstream target of β-catenin, and based on the most recent study on non-small cell lung cancer, there remains the possibility that the transcriptional activation of NAT10 might be modulated by c-Myc in CRC [23]. Mechanistically, we observed that NAT10 could activate the Wnt/β-catenin pathway by acetylating KIF23 mRNA. However, we considered KIF23 mRNA as the most potential target of NAT10. Notwithstanding, there are other possible ac^4^C targets involved in cancers, especially CRC. Thus, further studies are still necessary. Besides, our focus was only on the role of NAT10 in regulating ac^4^C modification, implying that more protein interaction mechanisms in CRC remain to be explored.

Taken together, we explored the mechanism underlying NAT10-mediated ac^4^C modification in regulating CRC progression and observed that NAT10 could acetylate KIF23 mRNA, resulting in the activation of the Wnt/β-catenin pathway and leading to tumor proliferation and metastasis.

## Conclusions

Briefly, our findings demonstrated that NAT10 might mediate CRC progression through ac^4^C modification. And, for the first time, the NAT10/KIF23/GSK-3β loop was recognized to regulate the proliferation and metastasis of CRC. By the application of remodelin, NAT10 seemed to be a potential therapeutic target for CRC (Fig. 8G).

## Supplementary Information

**Additional file 1: Table S1.** The H-score of NAT10, KIF23 and β-catenin and the clinicopathological information.

**Additional file 2: Table S2.** Antibodies used in the present study.

**Additional file 3: Table S3.** Primers, shRNAs and probes used in this study.

**Additional file 4: Table S4.** Relevance analysis of β-catenin expression in CRC patients.

**Additional file 5: Table S5.** The mRNA level of NAT10 and KIF23 in 80 pairs of CRC tissues.

**Additional file 6: Figure S1.** The expression of NAT10 in public datasets and the location of NAT10 in CRC cells. A. The mRNA level of NAT10 according to TCGA and GEO datasets. B. The protein level of NAT10 according to CPTAC datasets. C. The diagram of the TMA. D. HE staining of the TMA. E. The correlation of NAT10 expression with clinical features according to the TMA. F. ac^4^C staining of the TMA. G and H. The location of NAT10 in CRC cells. All data are presented as mean±SD. *P < 0.05, ****P < 0.0001, ns. not significant.

**Additional file 7: Figure S2.** Knockdown of NAT10 inhibits the proliferation, migration and invasion of DLD-1 cells in vitro. A. Transfection efficiency of NAT10 in DLD-1 cells, detected by qRT-PCR, WB, and dot blot. B-D. CCK-8, colony formation, and EdU assays were performed to detect the proliferation of DLD-1 cells. E. The distribution of the cell cycle was detected by flow cytometry in DLD-1 cells. F. Cells were treated with the serum-free medium for 36 h. Flow cytometry was used to detect the apoptotic rates (LR+UR) of DLD-1 cells. G. Transwell and wound healing assays were used to detect the migration and invasion of DLD-1 cells. LR, early apoptotic cells; UR, terminal apoptotic cells. Data are shown as mean±SD of three independent experiments, *P < 0.05, **P < 0.01, ***P < 0.001, ****P < 0.0001, ns. not significant.

**Additional file 8: Figure S3.** Knockdown of NAT10 suppresses the tumor growth and metastasis of DLD-1 cells in vivo. A. Representative images of subcutaneous xenograft tumors (*n* = 5 for each group). The tumor volumes were measured every 5 days and the tumor weights were analyzed. B. HE and IHC staining of xenograft tumors. The expression of Ki67 was detected by IHC. C. Representative images and analysis of luminescence intensity in metastasis models (*n* = 5 for each group). D. Representative image and HE staining of metastatic tumors in the livers and lungs of mice. The number of metastases in livers or lungs was analyzed. All data are presented as mean±SD. **P < 0.01, ***P < 0.001.

**Additional file 9: Figure S4.** The profile of NAT10-modified genes in CRC cells. A. Volcano plots of differentially expressed genes identified by RNA-seq. B. Overlapping analysis of the significantly down-regulated genes in SW480 and DLD-1 cells identified by RNA-seq. C. Overlapping analysis of the genes in SW480 and DLD-1 cells identified by RNA-seq. D. GSEA analysis of the genes described in (C). E. The process of acRIP-seq. F. Overlapping analysis of the top 46 genes in SW480 and DLD-1 cells reranked by fold-enrichment in RIP-seq and acRIP-seq. G. Distribution of NAT10-binding regions and ac^4^C peaks on the mRNA of six potential direct targets of NAT10 visualized by IGV. H. The mRNA level of seven potential direct targets of NAT10 detected by qRT-PCR in 24 CRC tissues and matched adjacent tissues. Data are shown as mean±SD of three independent experiments, *P < 0.05, **P < 0.01, ns. not significant.

**Additional file 10: Figure S5.** NAT10 mediates the mRNA degradation of KIF23 in an ac^4^C-dependent way. A. Correlation analysis between the mRNA levels of NAT10 and KIF23 according to the TCGA or GEO datasets. B. KIF23 staining of the TMA. The expression of KIF23 was analyzed by IHC. C. Correlation analysis between the protein levels of NAT10 and KIF23 in the TMA. D. NAT10 RIP followed by qPCR in DLD-1 cells. E. acRIP followed by qPCR in DLD-1 cells. F. The interaction between NAT10 and KIF23 mRNA was analyzed by RIP-qPCR assay in DLD-1 cells with NAT10 knockdown. G. The relative levels of ac^4^C in KIF23 were tested by acRIP-qPCR in DLD-1 cells with NAT10 knockdown. H. Relative RNA and protein level of KIF23 in DLD-1 cells upon NAT10 knockdown. I. The luciferase activity for the reporter containing the promoter region of KIF23 upon NAT10 knockdown or overexpression. J. Schematic of the wild-type or mutant regions in the 3’UTR of KIF23 mRNA. The luciferase activity for the reporter containing the NAT10-binding region or mutant upon NAT10 knockdown in DLD-1 cells. K. Schematic presentation of KIF23 mRNA and the location of probes used for REMSA. L. Schematic representation of Flag-tagged wild-type (NAT10-wt) and mutant (NAT10-mut) NAT10 constructs. M. Schematic representation of HA-tagged wild-type (KIF23-wt) and mutant (KIF23-mut) KIF23 constructs. N. The mRNA stability was detected by qRT-PCR in DLD-1 cells with the addition of actinomycin D (5 μg/mL). O. The protein expression of KIF23 with the treatment of CHX (100 μg/mL) in DLD-1 cells upon NAT10 knockdown. Data are shown as mean±SD of three independent experiments, *P < 0.05, **P < 0.01, ***P < 0.001, ****P < 0.0001, ns. not significant.

**Additional file 11: Figure S6.** The NAT10/KIF23 axis regulates CRC cells by activating the Wnt/β-catenin pathway. A. GSEA analysis of NAT10 in TCGA and GEO dataset. B. β-catenin staining of the TMA. The expression of β-catenin was analyzed by IHC. C. The expression of GSK-3β, phosphorylated GSK-3β and β-catenin were determined by WB upon NAT10 knockdown in DLD-1 cells. D. The level of β-catenin in the cell nucleus and cytoplasm was determined by WB in DLD-1 cells. E. β-catenin nuclear translocation and KIF23 were detected by IF staining upon NAT10 knockdown in DLD-1 cells. F. IHC staining of xenograft tumors. The expression of β-catenin and KIF23 were detected by IHC. G. Transfection efficiency of KIF23 in CRC cells, detected by qRT-PCR and WB. H and I. CCK-8 and colony formation assays were performed in shNAT10-2 and oeKIF23 co-transfected SW480 and DLD-1 cells and in oeNAT10 and shKIF23-2 co-transfected HT-29 cells. Data are shown as mean±SD of three independent experiments. **P < 0.01, ***P < 0.001, ****P < 0.0001, ns. not significant.

**Additional file 12: Figure S7.** The NAT10/KIF23 axis regulates CRC cells. A. EdU assays were performed in shNAT10-2 and oeKIF23 co-transfected SW480 and DLD-1 cells and in oeNAT10 and shKIF23-2 co-transfected HT-29 cells. B and C. The flow cytometry of cell cycle and apoptosis were performed in shNAT10-2 and oeKIF23 co-transfected SW480 and DLD-1 cells and in oeNAT10 and shKIF23-2 co-transfected HT-29 cells. D. Transwell and wound healing assays were performed in shNAT10-2 and oeKIF23 co-transfected SW480 and DLD-1 cells and in oeNAT10 and shKIF23-2 co-transfected HT-29 cells. Data are shown as mean±SD of three independent experiments. *P < 0.05, ***P < 0.001, ****P < 0.0001, ns. not significant.

**Additional file 13: Figure S8.** The GSK-3β/NAT10/KIF23/GSK-3β loop is involved in the CRC progression. A. The expression of GSK-3β, phosphorylated GSK-3β, and β-catenin along with NAT10, KIF23, and the downstream genes of β-catenin (Cyclin D1, c-Myc, Survivin, and Bcl-xL) were detected by WB in relatively treated DLD-1 cells. B. NAT10 and KIF23 were detected by IF staining with or without the treatment of LiCl (20mmol/L) in DLD-1 cells for 48 h. C. RIP and acRIP followed by qPCR with or without the treatment of LiCl (20mmol/L) in DLD-1 cells for 48 h. D. The expression of NAT10, KIF23, GSK-3β, phosphorylated GSK-3β, and β-catenin were detected by WB with or without the treatment of LiCl (20mmol/L) in DLD-1 cells for 48 h. Data are shown as mean±SD of three independent experiments. *P < 0.05.

## Data Availability

The data in the current study are available from the corresponding author on reasonable request.

## Abbreviations

CRC: Colorectal cancer
NAT10: N-acetyltransferase 10
KIF23: Kinesin Family Member 23
GSK-3β: Glycogen Synthase Kinase 3 Beta
GAPDH: Glyceraldehyde-3-Phosphate Dehydrogenase
OS: Overall survival rate
CCK-8: Cell counting Kit-8
EdU: 5-Ethynyl-2’-deoxyuridine
IHC: Immunohistochemistry
IF: Immunofluorescence
qRT-PCR: Quantitative reverse transcription polymerase chain reaction
WB: Western blot
shRNA: Short hairpin RNA
RIP: RNA immunoprecipitation
acRIP: Acetylated RNA immunoprecipitation
UTR: Untranslated region
CHX: Cycloheximide
