# Supplementary material for: Acetyltransferase NAT10 regulates the Wnt/β-catenin signaling pathway to promote colorectal cancer progression via ac4C acetylation of KIF23 mRNA
Source: J Exp Clin Cancer Res. 2022 Dec 15;41:345. doi: 10.1186/s13046-022-02551-7 (PMC9753290; doi:10.1186/s13046-022-02551-7)
Supplement: Supplementary file 4 — Additional file 4: Table S4. Relevance analysis of β-catenin expression in CRC patients. [file 13046_2022_2551_MOESM4_ESM.docx]

| **Table S4. Relevance analysis of β-catenin expression in CRC patients.** | | | | |
| --- | --- | --- | --- | --- |
| **Varible** | **All patients** | **β-catenin** | | **P value** |
|  |  | **High** | **Low** |  |
| All Cases | 80 | 40 | 40 |  |
| Age (years) |  |  |  |  |
| <60 | 23 | 12 | 11 | 0.805 |
| ≥60 | 57 | 28 | 29 |  |
| Gender |  |  |  |  |
| Male | 50 | 24 | 26 | 0.644 |
| Female | 30 | 16 | 14 |  |
| Tumor site  Colon  Rectum  Tumor size (cm) | 35  45 | 23  17 | 12  28 | **0.013^*^** |
| <5 | 49 | 24 | 25 | 0.819 |
| ≥5 | 31 | 16 | 15 |  |
| TNM staging system |  |  |  |  |
| T1 + T2 | 33 | 12 | 21 | **0.041^*^** |
| T3 + T4 | 47 | 28 | 19 |  |
| Tumor stage |  |  |  |  |
| Stage I+II | 43 | 14 | 29 | **<0.001^*^** |
| Stage III+IV | 37 | 26 | 11 |  |
| Lymph node metastasis |  |  |  |  |
| No | 46 | 16 | 30 | **0.002^*^** |
| Yes | 34 | 24 | 10 |  |
| Vascular invasion |  |  |  |  |
| No | 61 | 27 | 34 | 0.066 |
| Yes | 19 | 13 | 6 |  |
| Nerve invasion |  |  |  |  |
| No | 66 | 32 | 34 | 0.556 |
| Yes | 14 | 8 | 6 |  |
| Distant metastasis |  |  |  | **0.014^*^** |
| No | 63 | 27 | 36 |  |
| Yes | 17 | 13 | 4 |  |
| CEA (ng/ml) |  |  |  |  |
| <5 | 49 | 23 | 26 | 0.491 |
| ≥5 | 31 | 17 | 14 |  |
|  |  |  |  |  |

NOTE: TNM tumour node metastasis. CEA carcinoembryonic antige

P <0.05 was considered signiﬁcant. The bold type represents P values smaller than 0.05
