# Supplementary figures and images for: Acetyltransferase NAT10 regulates the Wnt/β-catenin signaling pathway to promote colorectal cancer progression via ac4C acetylation of KIF23 mRNA
Source: J Exp Clin Cancer Res. 2022 Dec 15;41:345. doi: 10.1186/s13046-022-02551-7 (PMC9753290; doi:10.1186/s13046-022-02551-7)

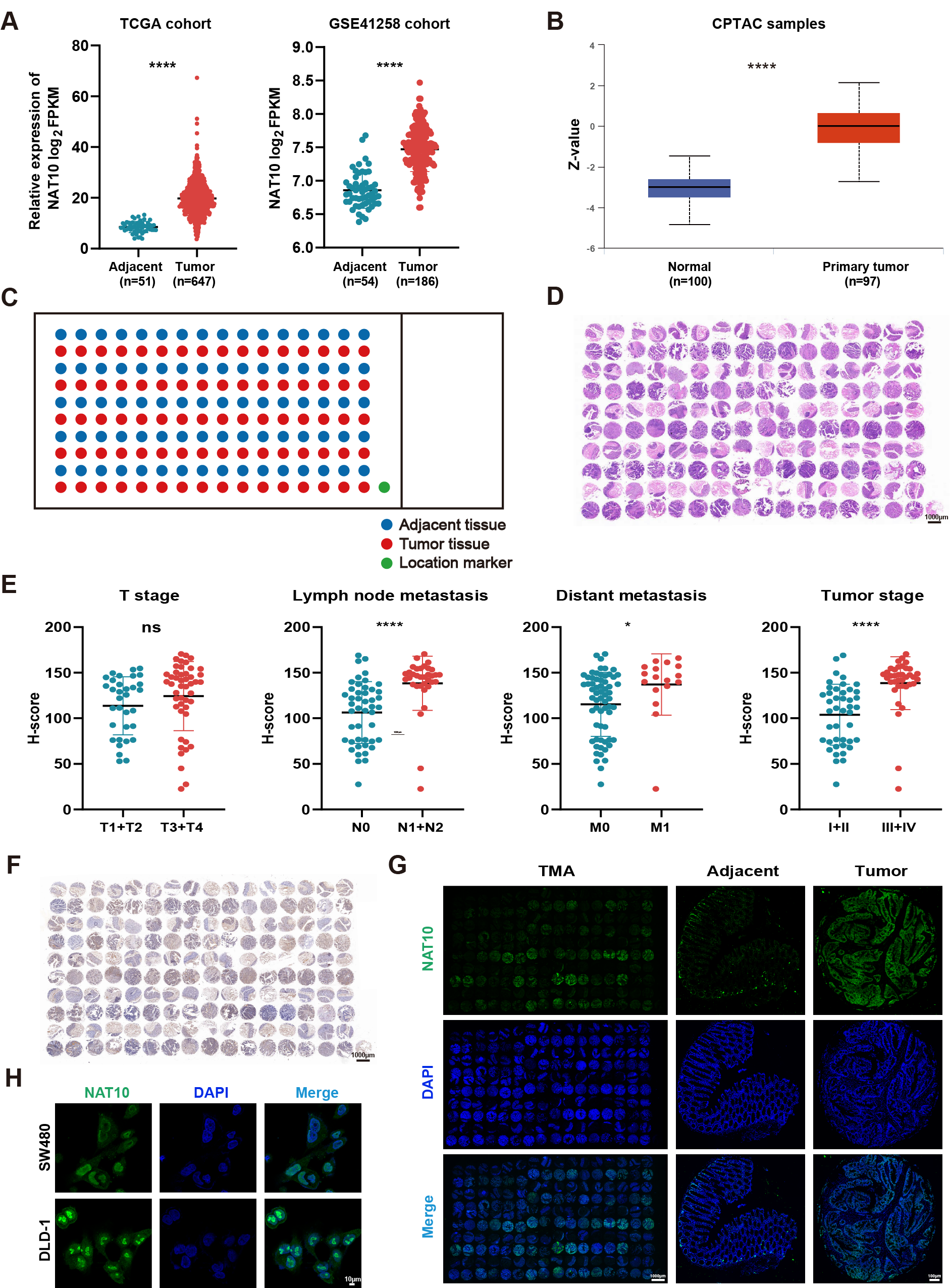

Supplement: Supplementary file 6 — Additional file 6: Figure S1. The expression of NAT10 in public datasets and the location of NAT10 in CRC cells. A. The mRNA level of NAT10 according to TCGA and GEO datasets. B. The protein level of NAT10 according to CPTAC datasets. C. The diagram of the TMA. D. HE staining of the TMA. E. The correlation of NAT10 expression with clinical features according to the TMA. F. ac4C staining of the TMA. G and H. The location of NAT10 in CRC cells. All data are presented as mean±SD. *P < 0.05, ****P < 0.0001, ns. not significant. [file 13046_2022_2551_MOESM6_ESM.tif]

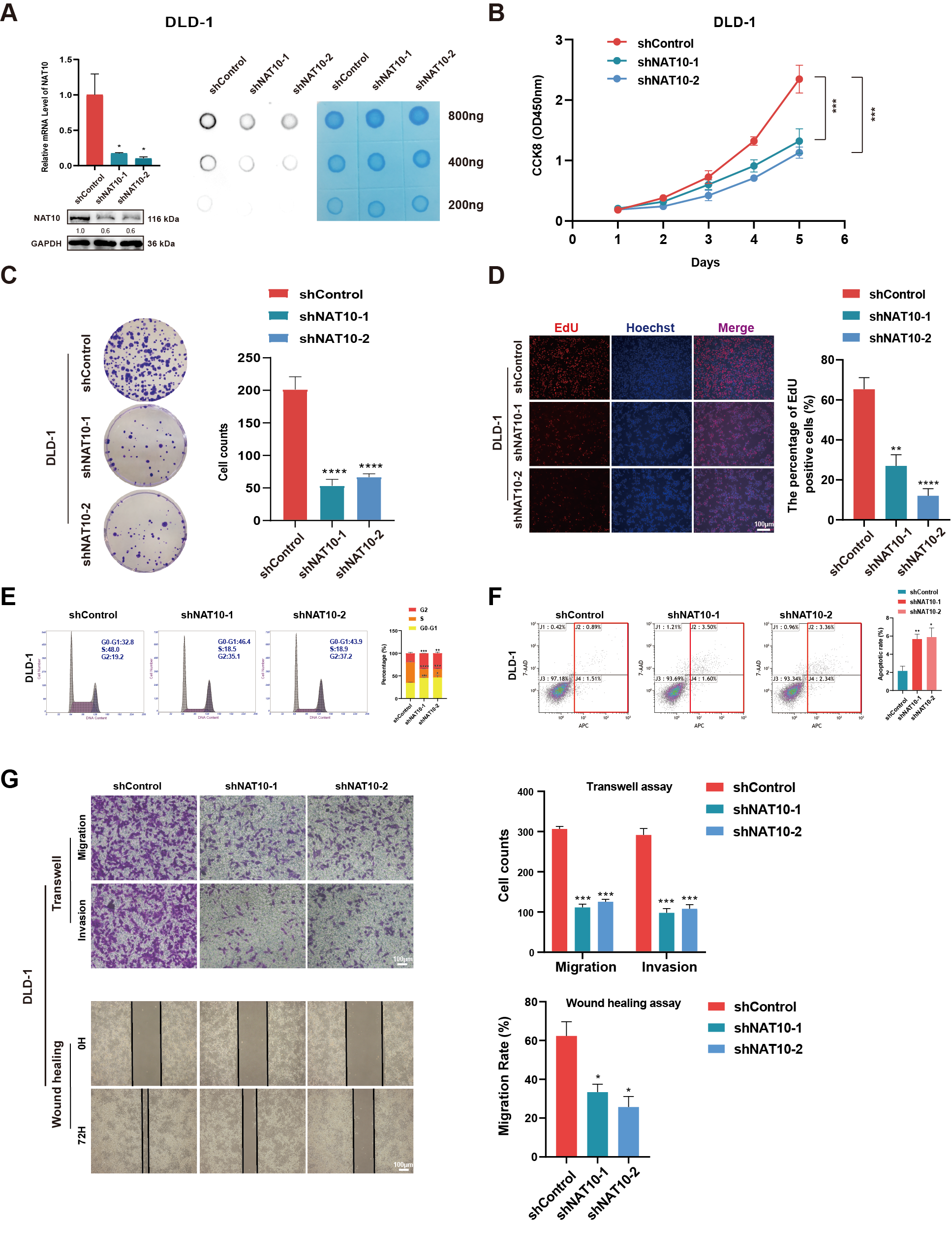

Supplement: Supplementary file 7 — Additional file 7: Figure S2. Knockdown of NAT10 inhibits the proliferation, migration and invasion of DLD-1 cells in vitro. A. Transfection efficiency of NAT10 in DLD-1 cells, detected by qRT-PCR, WB, and dot blot. B-D. CCK-8, colony formation, and EdU assays were performed to detect the proliferation of DLD-1 cells. E. The distribution of the cell cycle was detected by flow cytometry in DLD-1 cells. F. Cells were treated with the serum-free medium for 36 h. Flow cytometry was used to detect the apoptotic rates (LR+UR) of DLD-1 cells. G. Transwell and wound healing assays were used to detect the migration and invasion of DLD-1 cells. LR, early apoptotic cells; UR, terminal apoptotic cells. Data are shown as mean±SD of three independent experiments, *P < 0.05, **P < 0.01, ***P < 0.001, ****P < 0.0001, ns. not significant. [file 13046_2022_2551_MOESM7_ESM.tif]

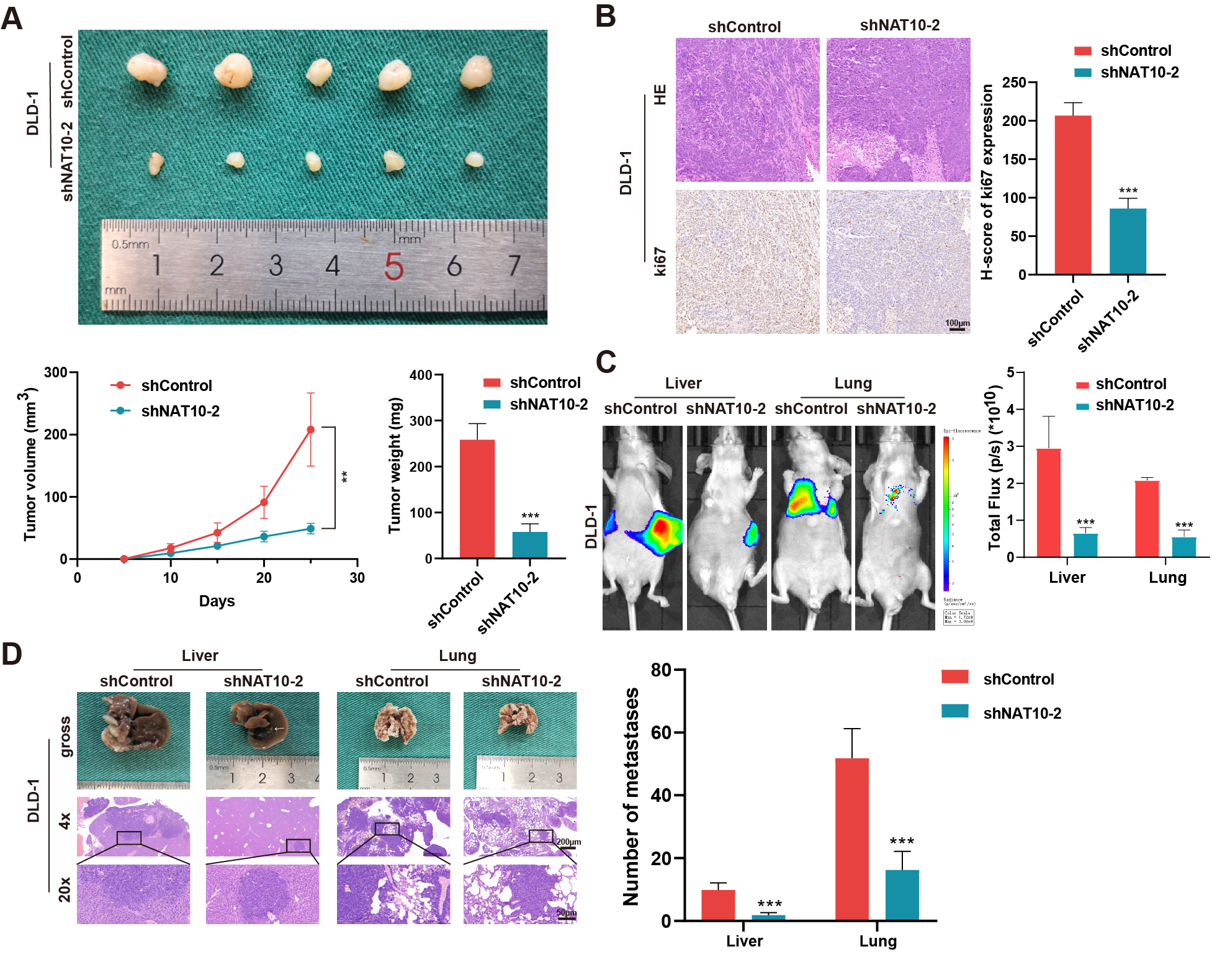

Supplement: Supplementary file 8 — Additional file 8: Figure S3. Knockdown of NAT10 suppresses the tumor growth and metastasis of DLD-1 cells in vivo. A. Representative images of subcutaneous xenograft tumors (n = 5 for each group). The tumor volumes were measured every 5 days and the tumor weights were analyzed. B. HE and IHC staining of xenograft tumors. The expression of Ki67 was detected by IHC. C. Representative images and analysis of luminescence intensity in metastasis models (n = 5 for each group). D. Representative image and HE staining of metastatic tumors in the livers and lungs of mice. The number of metastases in livers or lungs was analyzed. All data are presented as mean±SD. **P < 0.01, ***P < 0.001. [file 13046_2022_2551_MOESM8_ESM.tif]

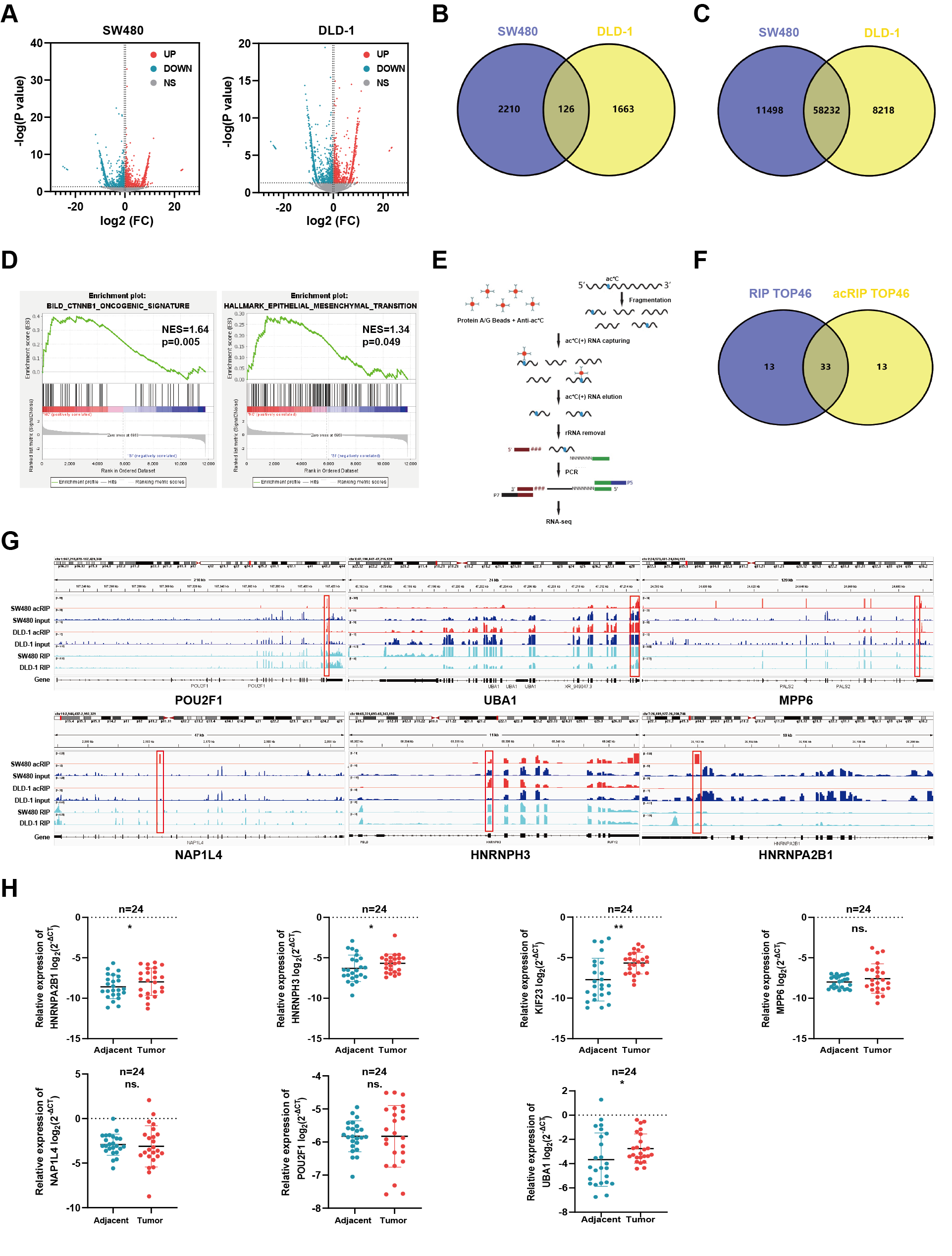

Supplement: Supplementary file 9 — Additional file 9: Figure S4. The profile of NAT10-modified genes in CRC cells. A. Volcano plots of differentially expressed genes identified by RNA-seq. B. Overlapping analysis of the significantly down-regulated genes in SW480 and DLD-1 cells identified by RNA-seq. C. Overlapping analysis of the genes in SW480 and DLD-1 cells identified by RNA-seq. D. GSEA analysis of the genes described in (C). E. The process of acRIP-seq. F. Overlapping analysis of the top 46 genes in SW480 and DLD-1 cells reranked by fold-enrichment in RIP-seq and acRIP-seq. G. Distribution of NAT10-binding regions and ac4C peaks on the mRNA of six potential direct targets of NAT10 visualized by IGV. H. The mRNA level of seven potential direct targets of NAT10 detected by qRT-PCR in 24 CRC tissues and matched adjacent tissues. Data are shown as mean±SD of three independent experiments, *P < 0.05, **P < 0.01, ns. not significant. [file 13046_2022_2551_MOESM9_ESM.tif]

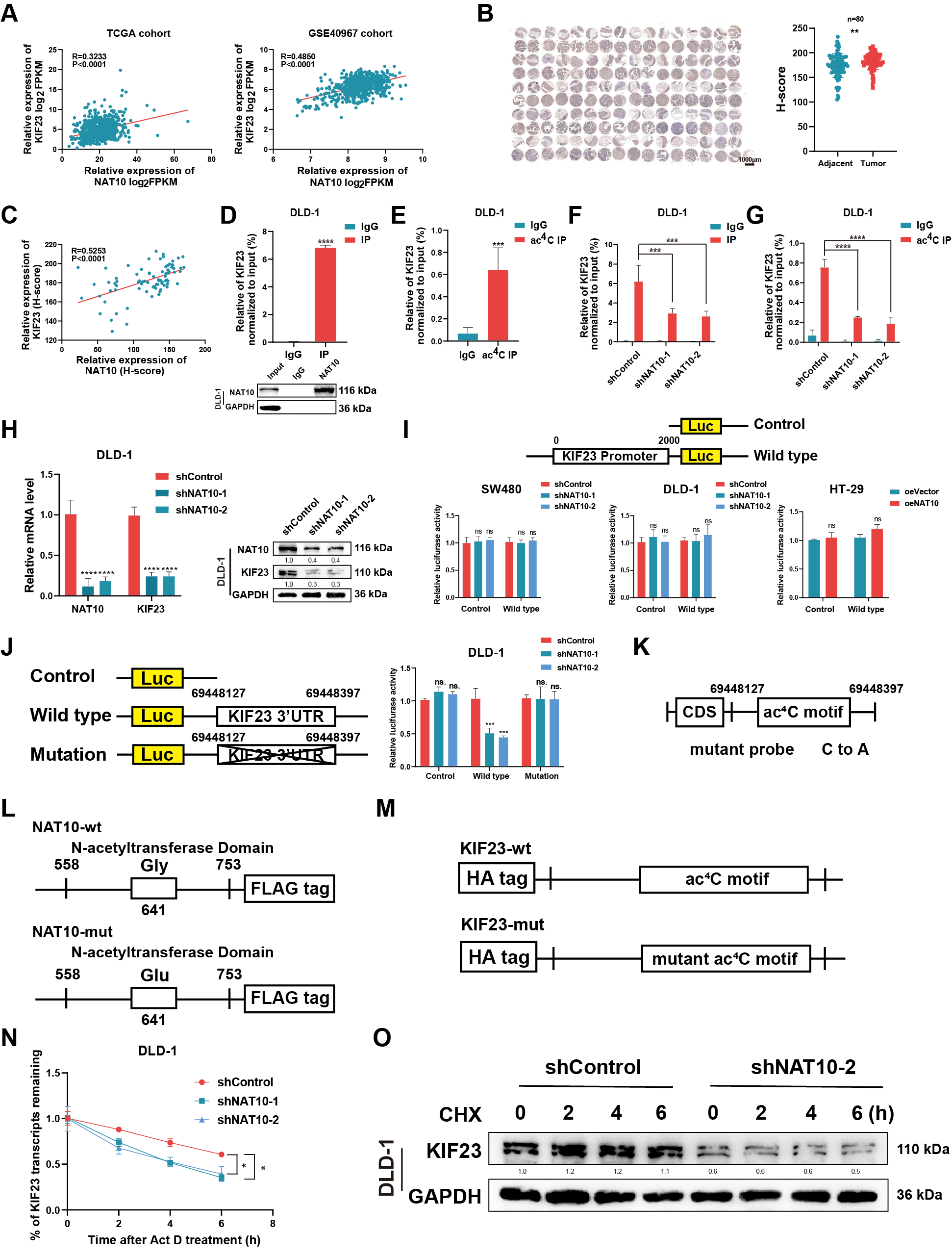

Supplement: Supplementary file 10 — Additional file 10: Figure S5. NAT10 mediates the mRNA degradation of KIF23 in an ac4C-dependent way. A. Correlation analysis between the mRNA levels of NAT10 and KIF23 according to the TCGA or GEO datasets. B. KIF23 staining of the TMA. The expression of KIF23 was analyzed by IHC. C. Correlation analysis between the protein levels of NAT10 and KIF23 in the TMA. D. NAT10 RIP followed by qPCR in DLD-1 cells. E. acRIP followed by qPCR in DLD-1 cells. F. The interaction between NAT10 and KIF23 mRNA was analyzed by RIP-qPCR assay in DLD-1 cells with NAT10 knockdown. G. The relative levels of ac4C in KIF23 were tested by acRIP-qPCR in DLD-1 cells with NAT10 knockdown. H. Relative RNA and protein level of KIF23 in DLD-1 cells upon NAT10 knockdown. I. The luciferase activity for the reporter containing the promoter region of KIF23 upon NAT10 knockdown or overexpression. J. Schematic of the wild-type or mutant regions in the 3’UTR of KIF23 mRNA. The luciferase activity for the reporter containing the NAT10-binding region or mutant upon NAT10 knockdown in DLD-1 cells. K. Schematic presentation of KIF23 mRNA and the location of probes used for REMSA. L. Schematic representation of Flag-tagged wild-type (NAT10-wt) and mutant (NAT10-mut) NAT10 constructs. M. Schematic representation of HA-tagged wild-type (KIF23-wt) and mutant (KIF23-mut) KIF23 constructs. N. The mRNA stability was detected by qRT-PCR in DLD-1 cells with the addition of actinomycin D (5 μg/mL). O. The protein expression of KIF23 with the treatment of CHX (100 μg/mL) in DLD-1 cells upon NAT10 knockdown. Data are shown as mean±SD of three independent experiments, *P < 0.05, **P < 0.01, ***P < 0.001, ****P < 0.0001, ns. not significant. [file 13046_2022_2551_MOESM10_ESM.tif]

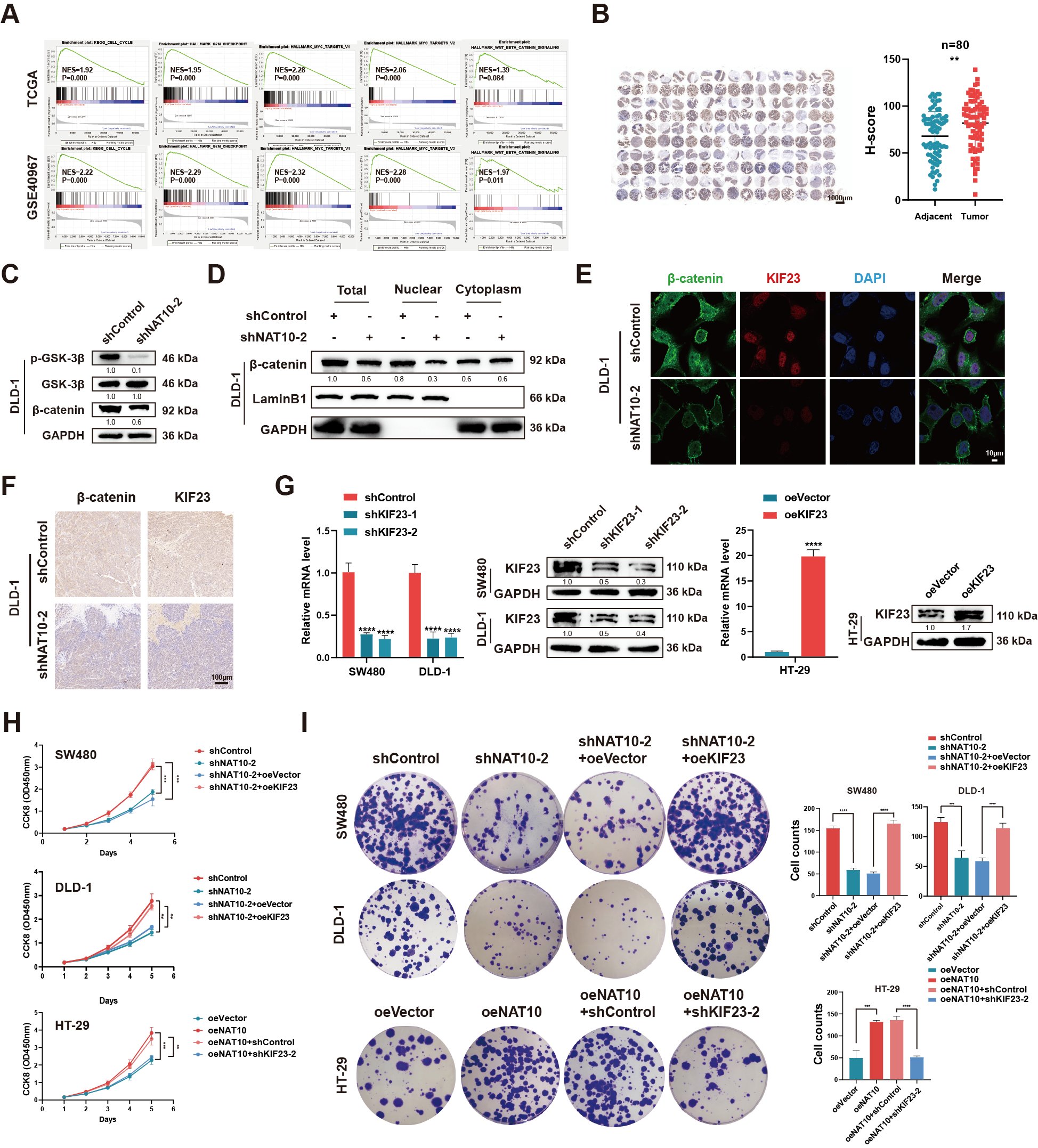

Supplement: Supplementary file 11 — Additional file 11: Figure S6. The NAT10/KIF23 axis regulates CRC cells by activating the Wnt/β-catenin pathway. A. GSEA analysis of NAT10 in TCGA and GEO dataset. B. β-catenin staining of the TMA. The expression of β-catenin was analyzed by IHC. C. The expression of GSK-3β, phosphorylated GSK-3β and β-catenin were determined by WB upon NAT10 knockdown in DLD-1 cells. D. The level of β-catenin in the cell nucleus and cytoplasm was determined by WB in DLD-1 cells. E. β-catenin nuclear translocation and KIF23 were detected by IF staining upon NAT10 knockdown in DLD-1 cells. F. IHC staining of xenograft tumors. The expression of β-catenin and KIF23 were detected by IHC. G. Transfection efficiency of KIF23 in CRC cells, detected by qRT-PCR and WB. H and I. CCK-8 and colony formation assays were performed in shNAT10-2 and oeKIF23 co-transfected SW480 and DLD-1 cells and in oeNAT10 and shKIF23-2 co-transfected HT-29 cells. Data are shown as mean±SD of three independent experiments. **P < 0.01, ***P < 0.001, ****P < 0.0001, ns. not significant. [file 13046_2022_2551_MOESM11_ESM.tif]

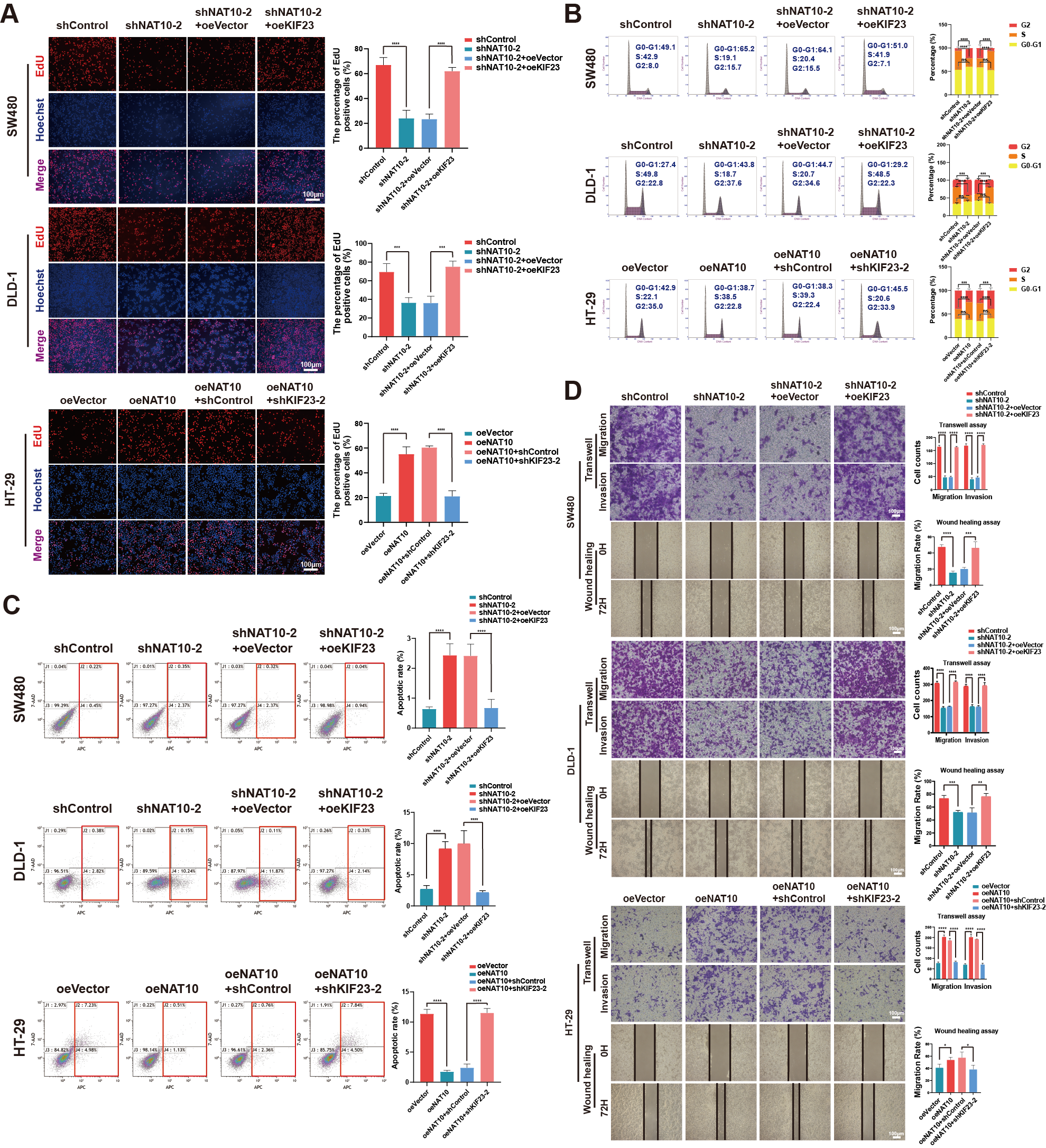

Supplement: Supplementary file 12 — Additional file 12: Figure S7. The NAT10/KIF23 axis regulates CRC cells. A. EdU assays were performed in shNAT10-2 and oeKIF23 co-transfected SW480 and DLD-1 cells and in oeNAT10 and shKIF23-2 co-transfected HT-29 cells. B and C. The flow cytometry of cell cycle and apoptosis were performed in shNAT10-2 and oeKIF23 co-transfected SW480 and DLD-1 cells and in oeNAT10 and shKIF23-2 co-transfected HT-29 cells. D. Transwell and wound healing assays were performed in shNAT10-2 and oeKIF23 co-transfected SW480 and DLD-1 cells and in oeNAT10 and shKIF23-2 co-transfected HT-29 cells. Data are shown as mean±SD of three independent experiments. *P < 0.05, ***P < 0.001, ****P < 0.0001, ns. not significant. [file 13046_2022_2551_MOESM12_ESM.tif]

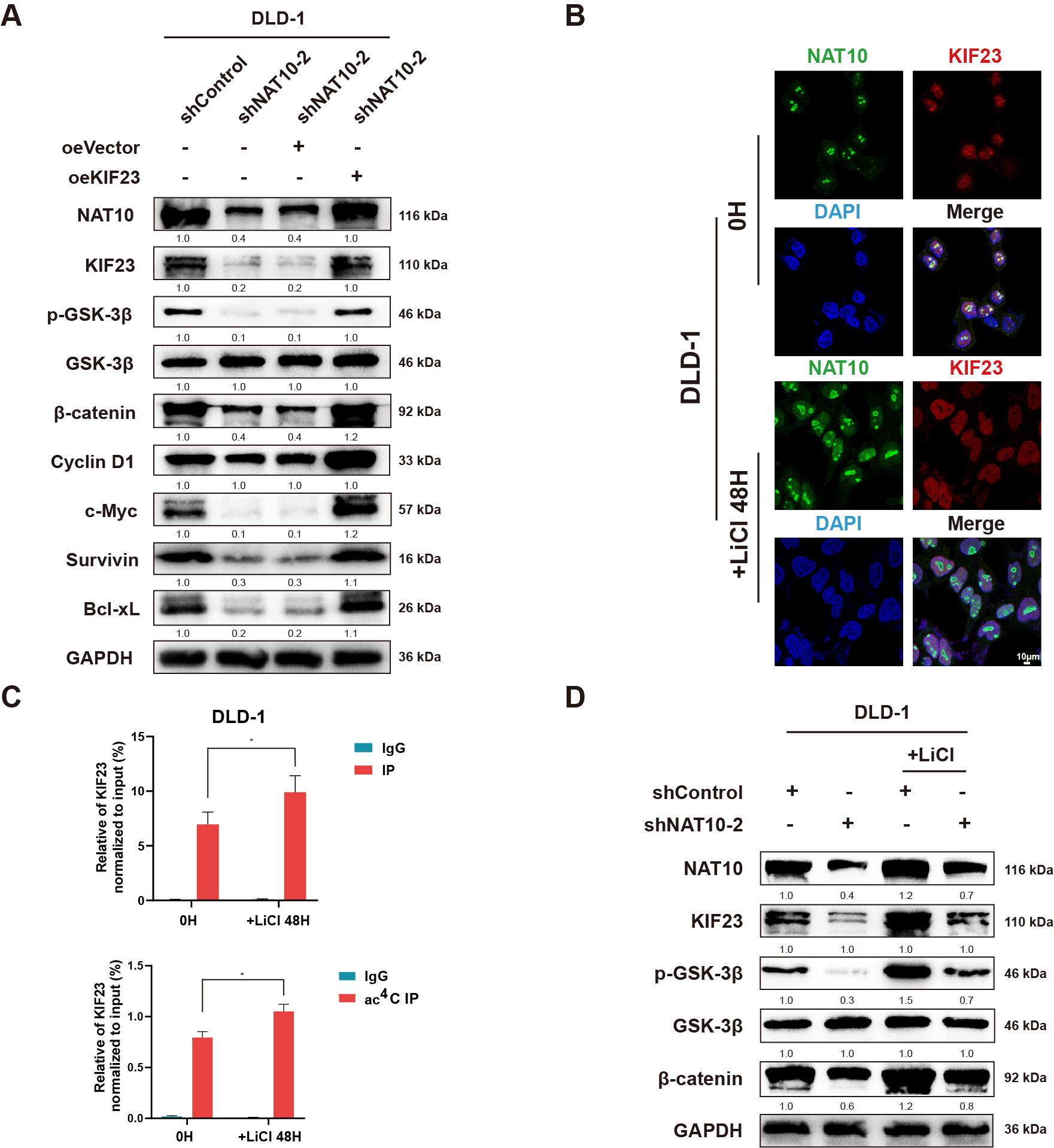

Supplement: Supplementary file 13 — Additional file 13: Figure S8. The GSK-3β/NAT10/KIF23/GSK-3β loop is involved in the CRC progression. A. The expression of GSK-3β, phosphorylated GSK-3β, and β-catenin along with NAT10, KIF23, and the downstream genes of β-catenin (Cyclin D1, c-Myc, Survivin, and Bcl-xL) were detected by WB in relatively treated DLD-1 cells. B. NAT10 and KIF23 were detected by IF staining with or without the treatment of LiCl (20mmol/L) in DLD-1 cells for 48 h. C. RIP and acRIP followed by qPCR with or without the treatment of LiCl (20mmol/L) in DLD-1 cells for 48 h. D. The expression of NAT10, KIF23, GSK-3β, phosphorylated GSK-3β, and β-catenin were detected by WB with or without the treatment of LiCl (20mmol/L) in DLD-1 cells for 48 h. Data are shown as mean±SD of three independent experiments. *P < 0.05. [file 13046_2022_2551_MOESM13_ESM.tif]
